# Aroxybutynin and atomoxetine (AD109) for obstructive sleep apnea: a randomized phase 3 trial (SynAIRgy)

**DOI:** 10.1093/ajrccm/aamag215

**Published:** 2026-05-18

**Authors:** Patrick J Strollo, Ron Farkas, Luigi Taranto-Montemurro, John Cronin, Sanjay R Patel, the SynAIRgy Investigators, Akinyemi Ajayi, Bernadette Alejandrino, Jerome Alonso, Najib Ayas, Francisco Badar, Jacob Coleman, William Cooper, Bruce Corser, Ronald Cridland, Dominick D'Aunno, Matthew Davis, Bertrand De Silva, Michael Downing, Alaa El-Gendy, Tomas Fiel, Steven Geller, James Geyer, Andrew Gould, Nella Green, Mario Guillen, Hermandeep Singh, John Hemmersmeier, John Hudson, Monica Jaffe, Thomas Jarrett, John Khoury, John Kimoff, Oleg Kouskov, Michael Lacey, Judith Leech, David Lesch, Michael Lillestol, Reinero Linares-Mera, Alan Lowe, Kinjal Madhav, David Marks, Ronald Mayfield, James Maynard, Jessica McCoun, Tatyana Miroshnikova, Rizwana Mohseni, Andrew Pastewski, Paresh Patel, Susheel Patil, Nirupa Paulraj, Enrique Pelayo, Dena Petersen, Alec Platt, Lew Pliamm, Bruce Rankin, Syed Raza, Anne Romaker, Mark Rosenthal, Eugene Ryan, Hector Sanchez, Andrew Schreiber, Sonja Schuetz, Sudhir Sehgal, Colin Shapiro, Craig Shapiro, Gerald Shockey, Sushil Singhi, Steve Sitar, Eileen Sloan, Damien Stevens, Kenneth Stiel, Masayoshi Takashima, Stephen Thein, Patrick Whitten, Paul Wylie, Dragos Zanchi, Brian Mangal, Brendan Lujan, Elaine Liu, Hoyee Leong, John Yee, Laura Gell, Huy Pho, Ana Sanchez-Azofra, Lori Eakes, Andrea Werner

**Affiliations:** Department of Medicine Service, Veteran Affairs Pittsburgh Healthcare System, Pittsburgh, PA, United States; Division of Pulmonary, Allergy, Critical Care and Sleep Medicine, Department of Medicine, University of Pittsburgh, Pittsburgh, PA, United States; Apnimed, Inc., Cambridge, MA, United States; Apnimed, Inc., Cambridge, MA, United States; Apnimed, Inc., Cambridge, MA, United States; Division of Pulmonary, Allergy, Critical Care and Sleep Medicine, Department of Medicine, University of Pittsburgh, Pittsburgh, PA, United States

**Keywords:** airway obstruction, hypoxia, patient-reported outcome measures

## Abstract

**Rationale:**

Many patients with obstructive sleep apnea (OSA) are unable to tolerate long-term positive airway pressure (PAP) therapy, highlighting the need for alternative treatments. AD109 (investigational fixed-dose oral combination of aroxybutynin 2.5 mg/atomoxetine 75 mg) is designed to target neuromuscular dysfunction in OSA.

**Objectives:**

To evaluate the efficacy/safety of AD109 over 6 months in a diverse OSA population unable to use PAP.

**Methods:**

SynAIRgy enrolled adults with mild-to-severe OSA who were intolerant to or refused PAP therapy into a randomized, double-blind, placebo-controlled, 26-week parallel-arm trial of AD109 vs placebo across 69 centers. The primary efficacy endpoint was change from baseline to week 26 in apnea–hypopnea index (AHI). Key secondary endpoints were oxygen desaturation index (ODI), Patient-Reported Outcomes Measurement Information System (PROMIS)–Fatigue T-score, hypoxic burden (HB), PROMIS-Sleep Impairment T-score, and proportion of participants with  ≥50% AHI reduction.

**Measurements and Main Results:**

A total of 646 eligible participants (median age 58 years, 49.3% female, median body mass index 32.4 kg/m^2^) were randomized. Median baseline AHI was 19.6 events/hour with 35% mild, 42% moderate, and 23% severe OSA. At week 26, mean AHI treatment difference was −4.0 events/hour (95% CI, −6.4 to −1.6; *P* = .001), representing a model-estimated 44.1% vs 17.6% decrease from baseline (*P* <.0001). AD109 demonstrated improvements in ODI and HB at week 26 vs placebo; however, no statistically significant difference was observed for PROMIS-Fatigue. Overall, 21.2% of participants on AD109 and 3.1% on placebo discontinued therapy due to adverse events. The most common adverse events with AD109 were dry mouth, nausea, insomnia, and urinary hesitation, with no serious treatment-related adverse events.

**Conclusions:**

AD109 significantly improved airway obstruction and oxygenation at 26 weeks across a broad range of patients unable to use PAP, suggesting that AD109 could become a potential treatment option for patients with OSA.

**Clinical trial registration:**

www.clinicaltrials.gov (NCT05813275 [SynAIRgy]).

At a Glance Commentary **Scientific Knowledge on the Subject:** AD109 (aroxybutynin 2.5 mg/atomoxetine 75 mg), an investigational, first-in-class, once-daily oral tablet, is designed to target the neuromuscular dysfunction underlying obstructive sleep apnea (OSA) by increasing upper airway muscle tone during sleep. In the phase 2b MARIPOSA trial, AD109 demonstrated significant improvement in the apnea–hypopnea index (AHI) in adults with mild-to-severe OSA over 4 weeks. An earlier phase 2 study suggested that AD109 may reduce AHI from the first dose. AD109 efficacy/safety beyond 4 weeks remain unknown.

**What This Study Adds to the Field:** SynAIRgy, a part of one of the largest clinical development efforts conducted for an OSA pharmacotherapy, is one of 2 phase 3 registrational trials evaluating the efficacy/safety of AD109. SynAIRgy is a randomized, double-blind, placebo-controlled, parallel-arm trial conducted in 646 participants with mild-to-severe OSA who were intolerant to or refused positive airway pressure therapy. SynAIRgy demonstrated significant improvements in airway obstruction and oxygenation across mild-to-severe OSA participants, supporting the potential for AD109 to be a new oral treatment option for patients with mild-to-severe OSA. Given existing OSA therapy limitations, AD109 may fill a large unmet medical need in providing patients with a pharmacologic therapy targeting the neuromuscular dysfunction underlying OSA pathogenesis.

Obstructive sleep apnea (OSA) is a chronic disease caused by sleep-related neuromuscular dysfunction in the setting of predisposing anatomic abnormalities. These pathophysiologic mechanisms lead to recurrent upper airway obstruction during sleep, resulting in chronic intermittent hypoxia and intrathoracic pressure swings that increase cardiac workload.^1^ Chronic intermittent hypoxia is associated with increased risk of long-term cardiometabolic and neurocognitive sequelae, and early mortality.^2-6^ OSA affects approximately 1 billion people worldwide, including approximately 80 million Americans.^7^^,^^8^ Up to 80% of individuals with OSA remain undiagnosed.^6^^,^^9-11^

Current treatment options for OSA, particularly positive airway pressure (PAP), can be clinically efficacious but are often poorly accepted or tolerated, leading to treatment refusal or low levels of adherence.^12-19^ Many individuals diagnosed with OSA who receive PAP treatment are undertreated or discontinue treatment within 1 year.^20^ Alternative treatment strategies, including oral appliances and upper airway surgery, are limited to particular populations, and these options also have limitations in acceptability and accessibility.^21^^,^^22^ Prevalence estimates indicate that less than 40% of patients with OSA are obese in community-based samples^23^^,^^24^; however, higher prevalence has been reported in the clinic setting.^25^ Weight reduction treatments can be helpful for patients with OSA and obesity, although many patients may experience residual OSA even after significant weight loss.^26-29^

AD109 is an investigational, once-daily-at-bedtime, fixed-dose oral combination of the novel antimuscarinic aroxybutynin (2.5 mg) with the selective norepinephrine reuptake inhibitor atomoxetine (75 mg) in one tablet as an anti-apneic neuromuscular modulator.^30-33^ It is designed to target the neuromuscular root cause of OSA through the synergy of aroxybutynin and atomoxetine, which together can increase upper airway muscle tone during sleep by stimulating the hypoglossal motor nucleus.^30-33^ Further details on the mechanism of action of AD109 have been recently published.^33^

In the phase 2b MARIPOSA trial (NCT05071612), AD109 demonstrated significant improvement in the apnea–hypopnea index based on 4% hypopnea desaturation (AHI) in adults with mild-to-severe OSA over a 4-week period.^31^ Earlier phase 2 studies (NCT04631107) suggested that AD109 may reduce AHI from the first night of dosing.^30^ We conducted the phase 3 SynAIRgy clinical trial to evaluate the efficacy, safety, and tolerability of AD109 vs placebo over 6 months in adults with mild-to-severe OSA who were intolerant to or refused PAP therapy. Some of the results of this study have been previously reported in the form of an abstract.^34^

## Methods

### Trial design

SynAIRgy (APC-APN-305; NCT05813275)^35^ was a phase 3, randomized, double-blind, placebo-controlled, 6-month parallel-arm study of AD109 (Figure S1), conducted at 69 sites in the United States and Canada in compliance with the Declaration of Helsinki and Good Clinical Practice guidelines outlined by the International Council for Harmonisation. Institutional review boards or independent ethics committees approved the protocol at each participating site (Online Supplement). Participants provided written informed consent before enrollment. This study followed the Consolidated Standards of Reporting Trials (CONSORT) reporting guideline.

### Participants

Patients aged  ≥18 years with mild-to-severe OSA who had failed or refused PAP therapy were screened for eligibility. Participants with AHI  ≥5 events/hour were initially enrolled. An apnea was defined by an event with  ≥90% reduction in airflow for  ≥10 seconds and hypopnea by  ≥30% reduction in airflow for  ≥10 seconds associated with a  ≥4% decrease in the oxygen saturation.^36^ The eligibility criterion of enrolling participants with AHI  ≥5 events/hour was subsequently changed to  ≥10 to ≤45 events/hour to avoid overrepresentation of participants with very severe or very mild OSA. Other inclusion criteria were an 8-item Patient-Reported Outcomes Measurement Information System (PROMIS)–Fatigue T-score >50, representing above-average fatigue and a body mass index (BMI) of 18.5 to 40 kg/m^2^ in men or 18.5 to 42 kg/m^2^ in women. Key exclusion criteria were clinically significant or medically uncontrolled cardiovascular disease, presence of narcolepsy, restless leg syndrome requiring medication, rapid eye movement (REM) sleep behavior disorder, bothersome symptoms of insomnia, and craniofacial malformation syndromes. Full inclusion and exclusion criteria are provided in the Supplementary Methods.

### Trial procedures

Eligible participants were randomized 1:1 to receive AD109 or placebo and stratified according to baseline AHI (<15, 15 to <30, ≥30 events/hour) and PROMIS-Fatigue T-score (<58.5 vs ≥58.5). Participants received AD109 or placebo during a 26-week period, including a 1-week run-in dose followed by 25 weeks at the target dose, then 2 weeks without dosing, followed by last visit at week 28 (Figure S1). AD109 dose was aroxybutynin 2.5 mg/atomoxetine 37.5 mg for week 1 and aroxybutynin 2.5 mg/atomoxetine 75 mg for the remaining weeks (dose reductions not permitted). The medication adherence (percent compliant) was calculated as medication adherence = 100 × (total number of doses received/total number of doses expected). Standard overnight polysomnography (PSG) was performed at baseline and weeks 4 and 26. Key patient-reported outcomes (PROs) were assessed using PROMIS-Fatigue and PROMIS-Sleep Impairment 8-item questionnaires at baseline and weeks 4, 13, and 26 (Supplementary Methods). Safety assessments included measurement of vital signs (heart rate and blood pressure) and monitoring of adverse events (AEs) and serious AEs. Vital signs were measured at baseline and weeks 4, 13, 26, and 28. AEs and serious AEs were actively collected from time of randomization until week 28.

An additional exploratory cohort of up to 100 participants receiving a concomitant glucagon-like peptide-1 (GLP-1) receptor agonist specifically for weight loss (ie, not for treatment of type 2 diabetes) was initiated and planned to be analyzed separately to evaluate the potential impact of weight loss on the efficacy and safety of AD109 compared with placebo. Due to low enrollment in this cohort, the prespecified exploratory analyses are not included in this report.

### Endpoints

The primary efficacy endpoint was the change in AHI from baseline to week 26. The key secondary efficacy endpoints (at week 26) were change from baseline in oxygen desaturation index based on 3% desaturation (ODI), PROMIS-Fatigue T-score, hypoxic burden based on 4% desaturation (HB), PROMIS-Sleep Impairment T-score, and proportion of participants with  ≥50% reduction in AHI. Prespecified exploratory endpoints (at week 26) included change from baseline in OSA severity category (none [AHI <5], mild [AHI 5 to <15], moderate [AHI 15 to <30], and severe [AHI  ≥30]), proportion of participants with AHI reduction of  ≥30% to  ≥90% by 10% increments, and proportion of breaths with snoring. Prespecified subgroup analyses were conducted for primary and secondary endpoints (Supplementary Methods). Safety was an additional endpoint.

### Statistical analysis

The sample size (640 participants) was calculated based on the number of participants required to provide >90% power for the primary endpoint and one of the key secondary efficacy endpoints (change from baseline to week 26 in PROMIS-Fatigue T-score) at a 2-sided significance level of 0.05 (Supplementary Methods). Primary analyses of the efficacy endpoints were performed using an intent-to-treat approach (ITT); supportive analyses were performed using the on-treatment estimand. The ITT analysis included all randomized participants who received  ≥1 dose of AD109/placebo and evaluated endpoints regardless of treatment adherence or discontinuation. The primary analysis for the primary and secondary endpoints defined on a continuous scale used a restricted maximum likelihood–based mixed model for repeated measurements in combination with the Newton–Raphson algorithm. For the ITT estimand, missing data were assumed to be missing-not-at-random, and endpoints were analyzed with a pattern-mixture model using a jump-to-reference approach by means of sequential modeling with multiple imputation.^37^ The on-treatment estimand included all randomized participants who received  ≥1 dose of AD109/placebo and who had  ≥1 post-baseline PSG assessment on treatment. The on-treatment estimand focused on endpoints observed during active treatment exposure and excluded data collected after treatment discontinuation, with missing values imputed using a last observation carried forward approach. Analysis of the proportion of participants with  ≥50% reduction in AHI at week 26 (key secondary endpoint not on a continuous scale) was conducted using the stratified Cochran–Mantel–Haenszel procedure, and estimates of risk difference between arms, along with the 2-sided 95% CI, were calculated based on the Cochran–Mantel–Haenszel stratum weights^38^ and the Sato variance estimator.^39^ For the proportion of participants with  ≥50% reduction in AHI, participants who discontinued treatment were considered nonresponders. For the primary endpoint, no adjustment for multiplicity was needed and a 2-sided 5% significance level was used. Adjustment for multiplicity of the key secondary endpoints was conducted through a closed testing approach, where each key secondary endpoint was tested in a prespecified order: (1) change from baseline to week 26 in ODI, (2) change from baseline to week 26 in PROMIS-Fatigue T-score, (3) change from baseline to week 26 in HB, (4) change from baseline to week 26 in PROMIS-Sleep Impairment T-score, and (5) proportion of participants with  ≥50% reduction in AHI at week 26. Key secondary endpoints that are nominally statistically significant but occur after a nonsignificant result in the prespecified hierarchical testing sequence should be interpreted with caution, as formal statistical significance cannot be claimed. Additional statistical details are provided in the Supplementary Methods. Safety was evaluated in the safety analysis set, including all randomized participants who received  ≥1 dose of AD109/placebo (Supplementary Methods). Participants with >1 event for the same AE were counted once.

## Results

### Participants

**Figure 1:**
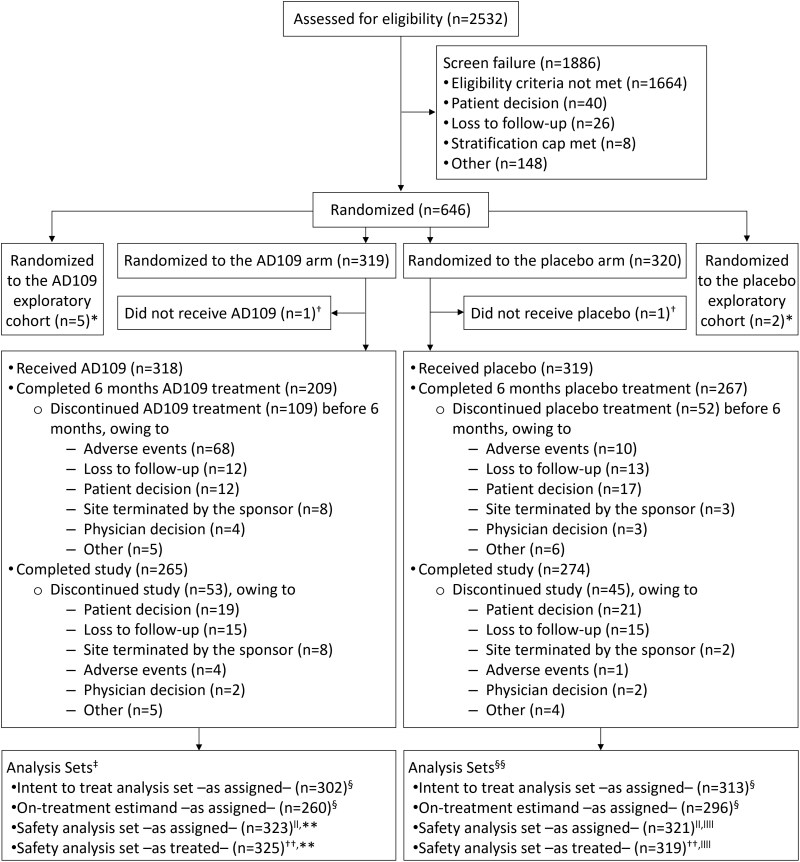
Screening, randomization, and follow-up in SynAIRgy participants. *The 7 participants (5 in the AD109 arm and 2 in the placebo arm) randomized and treated in the exploratory cohort are included in the safety analysis but not in the efficacy analyses. ^†^Reason of study discontinuation before receiving study medication was patient decision. ^‡^Sixteen participants enrolled at site 1416 in the AD106 arm were excluded from the efficacy analyses because the site was terminated due to Good Clinical Practice noncompliance. ^§^These numbers reflect participants by assigned treatment (2 participants assigned to placebo, received AD109 instead), and the intent-to-treat and on-treatment estimands were performed based on the assigned treatment. ^‖^These numbers reflect participants by assigned treatment, but 2 participants who were assigned to placebo received AD109 instead. **Includes 5 participants enrolled and treated in the exploratory cohort and 16 participants enrolled at site 1416. ^††^These numbers reflect participants by actual treatment and were used for the safety analysis. ^§§^Six participants enrolled at site 1416 in the placebo arm were excluded from the efficacy analyses because the site was terminated due to Good Clinical Practice noncompliance. ^‖‖^Includes 2 participants enrolled and treated in the exploratory cohort and 6 participants enrolled at site 1416.

Between December 20, 2023 and March 26, 2025, 646 participants with mild-to-severe OSA were enrolled and randomized to receive AD109 (*n* = 324) or placebo (*n* = 322) (Figure 1). In the main cohort, a total of 639 participants were enrolled and randomized to receive AD109 (*n* = 319) or placebo (*n* = 320) (Figure 1).

Among participants who received treatment, 84.6% (539/637) completed the trial, with 83.3% (265/318) in the AD109 group and 85.9% (274/319) in the placebo group. Overall, 74.7% (476/637) completed 6 months of treatment (65.7% [209/318] with AD109 and 83.7% [267/319] with placebo; Figure 1). In the AD109 arm, treatment adherence (percent compliant) was 95.7% (SD, 9.2%) for participants completing the study on treatment, and 90.2% (SD, 35.2%) for those discontinuing treatment. In the placebo arm, treatment adherence was 95.5% (SD, 9.5%) for participants completing the study on treatment, and 77.5% (SD, 42.4%) for those discontinuing treatment.

Participants’ demographics and disease characteristics were well balanced between the treatment arms and reflective of the adult patient population with OSA (Table 1). At baseline, the median BMI was 32.4 kg/m^2^ (minimum-maximum: 18.5-42.0), median age was 58 years (minimum-maximum: 24-87), and median AHI was 19.6 events/hour (minimum-maximum: 5.1-101.8) (Table 1). The distribution of baseline OSA severity was broad and right-skewed with 34.8% (214/615) mild, 42.1% (259/615) moderate, and 23.1% (142/615) severe (Table 1, Figure S2). There was a balanced proportion of female and male participants, and the racial representation in this study was comparable to that of the US adult population^40^ (Table 1).

**Table 1:** Baseline demographics and clinical characteristics of the SynAIRgy participants.

Characteristic	**AD109 (*n*** = **302)**	**Placebo (*n*** = **313)**	**Total (*N*** = **615)**
**Age, y, median (minimum-maximum)**	59 (24-83)	58 (25-87)	58 (24-87)
**Sex, *n* (%)**			
**Male**	152 (50.3)	160 (51.1)	312 (50.7)
**Female**	150 (49.7)	153 (48.9)	303 (49.3)
**Race, *n* (%)**			
**American Indian or Alaska Native**	2 (0.7)	2 (0.6)	4 (0.7)
**Asian**	23 (7.6)	22 (7.0)	45 (7.3)
**Black or African American**	55 (18.2)	62 (19.8)	117 (19.0)
**Native Hawaiian or Other Pacific Islander**	3 (1.0)	1 (0.3)	4 (0.7)
**White**	212 (70.2)	216 (69.0)	428 (69.6)
**Other**	2 (0.7)	3 (1.0)	5 (0.8)
**Multiple**	3 (1.0)	5 (1.6)	8 (1.3)
**Not reported/unknown**	2 (0.7)	2 (0.6)	4 (0.7)
**Ethnicity, *n* (%)**			
**Hispanic or Latino**	63 (20.9)	69 (22.0)	132 (21.5)
**Not Hispanic or Latino**	237 (78.5)	243 (77.6)	480 (78.0)
**Not reported/unknown**	2 (0.7)	1 (0.3)	3 (0.5)
**Body weight, kg, mean (SD)**	93.3 (17.0)	93.1 (17.1)	93.2 (17.0)
**BMI, kg/m^2^, median (minimum-maximum)**	32.5 (18.5-41.9)	32.4 (20.1-42.0)	32.4 (18.5-42.0)
**BMI distribution, *n* (%)**			
**18.5-29.9 kg/m^2^**	107 (35.4)	102 (32.6)	209 (34.0)
**30-34.9 kg/m^2^**	100 (33.1)	111 (35.5)	211 (34.3)
**≥35 kg/m^2^**	95 (31.5)	100 (31.9)	195 (31.7)
**AHI, events/h, median (minimum-maximum)**	19.8 (5.2-101.8)	19.1 (5.1-70.0)	19.6 (5.1-101.8)
**OSA severity, *n* (%)**			
**AHI 5 to <15 events/h (mild)**	105 (34.8)	109 (34.8)	214 (34.8)
**AHI 15 to <30 events/h (moderate)**	125 (41.4)	134 (42.8)	259 (42.1)
**AHI ≥30 events/h (severe)**	72 (23.8)	70 (22.4)	142 (23.1)
**ODI, events/h, median (minimum-maximum)**	27.3 (6.8-96.3)	26.9 (4.5-79.3)	27.0 (4.5-96.3)
**HB, %min/h, median (minimum-maximum)**	33.7 (0.8-379.8)	28.8 (2.4-272.8)	31.1 (0.8-379.8)
**PROMIS-Fatigue T-score, mean (SD)**	59.2 (7.0)	58.9 (6.7)	59.0 (6.8)
**PROMIS-Sleep Impairment T-score, mean (SD) [*n*]**	58.9 (7.2) [299]	58.6 (7.4) [309]	58.7 (7.3) [608]
**ESS score, mean (SD) [*n*]**	10.0 (4.6) [299]	10.1 (4.7) [311]	10.0 (4.6) [610]
**PAP intolerance, *n* (%)**	208 (68.9)	203 (64.9)	411 (66.8)
**PAP refusal, *n* (%)**	94 (31.1)	111 (35.5)	205 (33.3)
**Hypertension, *n* (%)**	152 (50.3)	153 (48.9)	305 (49.6)
**Diabetes^a^, *n* (%)**	52 (17.2)	51 (16.3)	103 (16.7)
**Hyperlipidemia^b^, *n* (%) **	116 (38.4)	117 (37.4)	233 (37.9)
**Gastroesophageal reflux disease, *n* (%)**	54 (17.9)	56 (17.9)	110 (17.9)
**Mood disturbances^c^, *n* (%)**	60 (19.9)	45 (14.4)	105 (17.1)
**Insomnia^d^, *n* (%)**	17 (5.6)	15 (4.8)	32 (5.2)

Abbreviations: AHI, apnea–hypopnea index based on 4% hypopnea desaturation; BMI, body mass index; ESS, Epworth Sleepiness Scale; HB, hypoxic burden based on 4% desaturation; ODI, oxygen desaturation index based on 3% desaturation; OSA, obstructive sleep apnea; PAP, positive airway pressure; PROMIS, Patient-Reported Outcomes Measurement Information System; SD, standard deviation.

a Includes type 2 diabetes mellitus, diabetes mellitus, and type 1 diabetes mellitus.

b Includes hypercholesterolemia, hypertriglyceridemia, hyperlipidemia, dyslipidemia, blood cholesterol increased, blood triglycerides increased, and type V hyperlipidemia.

c Includes depression, major depression, anxiety, social anxiety disorder, panic attack, and mood swings.

d Baseline insomnia was assessed based on participant-reported medical history obtained at screening and includes initial insomnia (difficulty falling asleep at the beginning of the night), insomnia, and middle insomnia (difficulty maintaining sleep, characterized by frequent or prolonged nighttime awakenings).

### Efficacy

**Figure 2:**
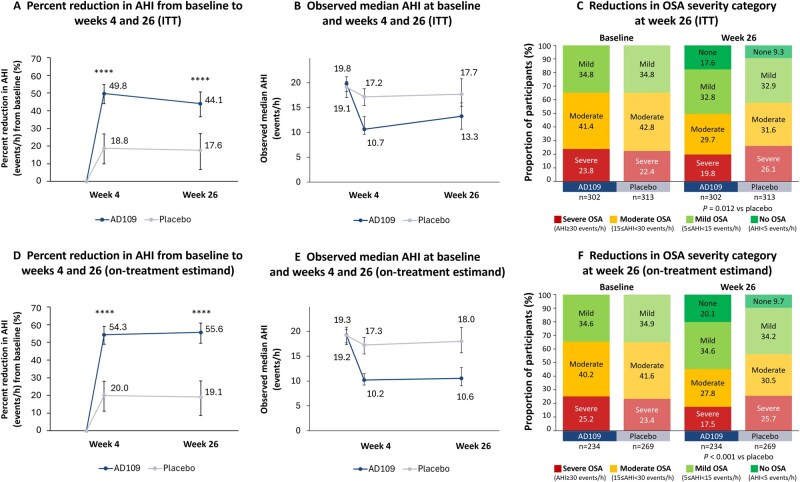
Reductions in AHI with AD109 as compared with placebo at weeks 4 and 26 and disease severity classification at week 26 (primary and exploratory endpoints). Model-generated geometric mean percent reduction in AHI from baseline to weeks 4 and 26 for the ITT (A) and on-treatment estimands (D). Observed median AHI at baseline to weeks 4 and 26 for the ITT (B) and on-treatment estimand (E). Proportion of participants with change in OSA severity category from baseline to week 26 in the ITT (C) and on-treatment estimands (F). In (A) and (C), due to skewed distribution, AHI was analyzed and modeled on the natural log scale; least squares mean estimated relative change from baseline from mixed model for repeated measurements analysis were back-transformed to original scale of relative change from baseline and expressed as a percentage for presentation. Log (0.01) was used if AHI was reported as 0. Error bars in (A, B, D, and E) represent 95% CIs. ****P <.0001 vs placebo. Abbreviations: AHI, apnea–hypopnea index based on 4% desaturation; CI, confidence interval; ITT, intent to treat; OSA, obstructive sleep apnea.

Mean change in AHI from baseline to week 26 was −3.3 events/hour (95% CI, −5.1 to −1.5) in the AD109 arm and 0.7 events/hour (95% CI, −1.0 to 2.4) in the placebo arm, for an estimated mean treatment difference of −4.0 events/hour (95% CI, −6.4 to −1.6; *P* = .001; Table 2). These changes represent a model-estimated 44.1% geometric mean reduction in AHI from baseline to week 26 in the AD109 arm vs 17.6% reduction in the placebo arm (*P* <.0001; Figure 2A). AD109 improved AHI as early as week 4 (Figure 2A). The median (95% CI) AHI was reduced from 19.8 (18.3-21.2) in the moderate severity range at baseline to 13.3 (10.7-15.9) in the mild severity range at week 26 with AD109, and from 19.1 (17.0-20.2) at baseline to 17.7 (15.3-20.8) at week 26 with placebo (Figure 2B; Table S1). The change in median AHI closely mirrored the percent change, estimated in the statistical model using geometric means. Similar improvements in AHI at week 26 were observed among participants in the AD109 arm across various subgroups including sex, baseline OSA severity categories (mild, moderate, and severe), and BMI classes (Figure S3). AD109 treatment effects on AHI were consistent across estimands, with larger effect sizes observed in the supportive on-treatment estimand in participants who tolerated treatment (Table 3, Figure 2D and E, Figure S3).

**Table 2:** Effect of AD109 on primary and key secondary efficacy endpoints (ITT).

**ITT (N = 615)**	**AD109 (n = 302)**	**Placebo (n = 313)**	**Estimated treatment difference or adjusted risk difference and P value** ^a^
**Primary efficacy endpoint**			
**Change in AHI from baseline to week 26, LS mean (95% CI), events/h**	−3.3 (−5.1 to −1.5)	0.7 (−1.0 to 2.4)	−4.0^b^ (−6.4 to −1.6)
			*P* = .001
**Key secondary efficacy endpoints**			
**Change in ODI from baseline to week 26, LS mean (95% CI), events/h**	−3.7 (−5.6 to −1.7)	0.9 (−1.1 to 2.8)	−4.5 (−7.3 to −1.8)
			*P* = .001
**Change in PROMIS-Fatigue T-score from baseline to week 26, LS mean (95% CI)**	−7.4 (−8.3 to −6.5)	−6.2 (−7.1 to −5.3)	−1.2 (−2.4 to 0.0)
			*P* = .059
**Percent reduction in geometric mean^c^ in HB from baseline to week 26, LS mean (95% CI), %min/h**	44.7 (34.5-53.4)	8.5 (‒8.1 to 22.5)	39.6 (23.6-52.3)
			*P* <.0001^d^
**Change in PROMIS-Sleep Impairment T-score from baseline to week 26, LS mean (95% CI)**	−7.6 (−8.5 to −6.6)	−6.9 (−7.8 to −5.9)	−0.7 (−2.0 to 0.7)
			*P* = .316
**Participants with ≥50% reduction in AHI at week 26, *n* (%)**	78 (25.8)	62 (19.8)	5.7 (−1.0 to 12.4)^e^ ^,^ ^f^
			*P* = .182

Abbreviations: AHI, apnea–hypopnea index based on 4% hypopnea desaturation; CI, confidence interval; HB, hypoxic burden based on 4% desaturation; ITT, intent to treat; LS, least squares; ODI, oxygen desaturation index based on 3% desaturation; PROMIS, Patient-Reported Outcomes Measurement Information System.

a Differences between treatment arms are presented as estimated treatment difference (AD109 − placebo) with the exception of HB, which was log transformed first to provide a geometric mean from baseline to week 26 (AD109/placebo); adjusted risk difference is shown for participants with  ≥50% reduction in AHI at week 26.

b The placebo-adjusted reduction at week 4 was −5.7 (−7.5 to −3.8); *P* <.0001.

c HB was analyzed on the natural log scale, then back-transformed to original scale of relative change from baseline and expressed as a percentage. Log (0.01) was used if HB was reported as 0.

d Represents nominal *P* value.

e The treatment difference at week 4 was 21.7 (14.8-28.5); *P* <.0001.

f Analysis approach in which any participant off treatment was treated as a nonresponder.

**Table 3:** Effect of AD109 on primary and key secondary efficacy endpoints (on-treatment estimand).

**On-treatment estimand (N = 556)**	**AD109 (n = 260)**	**Placebo (n = 296)**	**Estimated treatment difference or adjusted risk difference and P value** ^a^
**Primary efficacy endpoint**			
**Change in AHI from baseline to week 26, LS mean (95% CI), events/h**	−6.1 (−7.8 to −4.4)	0.4 (−1.2 to 2.0)	−6.5^b^ (−8.8 to −4.3)
			*P* <.0001
**Key secondary efficacy endpoints**			
**Change in ODI from baseline to week 26, LS mean (95% CI), events/h**	−6.5 (−8.4 to −4.6)	0.7 (−1.1 to 2.5)	−7.2 (−9.8 to −4.7)
			*P* <.0001
**Change in PROMIS-Fatigue T-score from baseline to week 26, LS mean (95% CI)**	−7.5 (−8.4 to −6.6)	−6.2 (−7.0 to −5.3)	−1.4 (−2.6 to −0.1)
			*P* = .030
**Percent reduction in geometric mean^c^ in HB from baseline to week 26, LS mean (95% CI), %min/h**	60.5 (52.8-66.9)	14.7 (‒0.7 to 27.8)	53.6 (41.0-63.6)
			*P* <.0001
**Change in PROMIS-Sleep Impairment T-score from baseline to week 26, LS mean (95% CI)**	−7.6 (−8.6 to −6.7)	−6.8 (−7.7 to −5.9)	−0.8 (−2.2 to 0.5)
			*P* = .231
**Participants with ≥50% reduction in AHI at week 26, *n* (%)**	103 (39.6)	66 (22.3)	17.3^d^ (9.6-25.0)
			*P* <.0001

Abbreviations: AHI, apnea–hypopnea index based on 4% hypopnea desaturation; CI, confidence interval; HB, hypoxic burden based on 4% desaturation; LS, least squares; ODI, oxygen desaturation index based on 3% desaturation; PROMIS, Patient-Reported Outcomes Measurement Information System.

a Differences between treatment arms are presented as estimated treatment difference (AD109-placebo) with the exception of HB which was log transformed first to provide a geometric mean from baseline to week 26 (AD109/placebo); adjusted risk difference is shown for participants with 50% reduction in AHI at week 26.

b The placebo-adjusted reduction at week 4 was −6.7 (−8.7 to −4.8); *P* <.0001.

c HB was analyzed on the natural log scale, then back-transformed to original scale of relative change from baseline and expressed as a percentage. Log (0.01) was used if HB was 0.

d The treatment difference at week 4 was 27.1 (19.7-34.4); *P* <.0001.

A significant difference in ODI was observed between treatment arms. Participants in the AD109 arm had a mean reduction in ODI at week 26 of 3.7 events/hour vs mean increase of 0.9 events/hour for participants in the placebo arm (*P* = .001; Table 2). AD109 treatment effects on ODI were consistent across estimands, with larger effect sizes observed in the supportive on-treatment estimand (Table 3).

AD109 reduced PROMIS-Fatigue T-scores at week 26 by a mean of 7.4 points compared with 6.2 points in the control arm, such that the difference between arms did not meet statistical significance (*P* = .059; Table 2). The impact of AD109 on PROMIS-Fatigue T-score at week 26 was more pronounced in participants who were more fatigued (*P* = .049) or had more daytime sleepiness (*P* = .007) at baseline (Figure S6). In the supportive on-treatment estimand analyses, PROMIS-Fatigue T-scores fell at week 26 by 7.5 points with AD109 vs 6.2 points with placebo (*P* = .030; Table 3).

Based on the prespecified hierarchical testing approach for secondary endpoints, formal endpoint testing ended before analysis of HB. The geometric mean percent reduction in HB at week 26 was 44.7% for participants in the AD109 arm vs 8.5% for participants in the placebo arm (nominal *P* <.0001; Table 2). AD109 improved HB as early as week 4 (Figure S4). Similar improvements in HB at week 26 were observed among participants in the AD109 arm across various subgroups (Figure S5). AD109 treatment effects on HB were consistent across estimands, with larger effect sizes observed in the supportive on-treatment estimand (Table 3, Figures S4 and S5).

The PROMIS-Sleep Impairment T-score improved by 7.6 points in the AD109 arm but the improvement in the placebo arm was similar, such that no treatment effect was observed between arms (*P* = .316; Table 2).

The proportion of participants with reduction in AHI  ≥50% at week 26 was not statistically significant between participants in the AD109 arm (25.8%) vs placebo (19.8%, *P* = .182; Table 2). However, in the supportive on-treatment estimand analyses, a higher proportion of participants in the AD109 arm achieved  ≥50% reduction in AHI vs placebo (39.6% vs 22.3%; *P* <.0001; Table 3).

**Figure 3:**
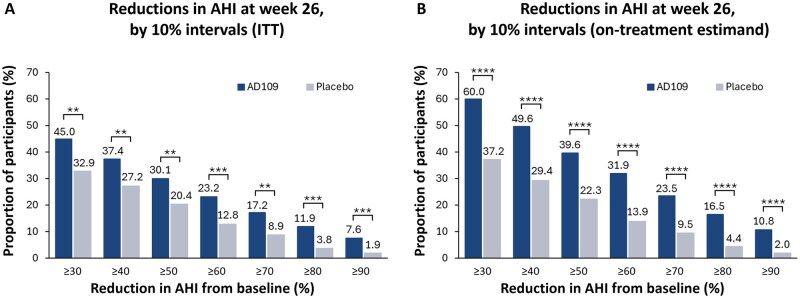
AHI responder analysis with AD109 as compared with placebo (exploratory endpoints). Proportion of participants with AHI reduction of ≥30% to  ≥90% at week 26 in the ITT (A) and the on-treatment estimands (B). (A) is a supportive analysis in which missing data were treated as nonresponders; observed data off treatment were included. ****P <.0001 vs placebo. ***P <.001 vs placebo. **P <.01 vs placebo. Abbreviations: AHI, apnea–hypopnea index based on 4% desaturation; ITT, intent to treat.

AD109 improved OSA disease severity category in 41.8% of participants (vs 33.6% for placebo), with 17.6% achieving complete OSA disease control (AHI <5) at week 26 vs 9.3% for placebo (*P* = .012; Figure 2C). The proportion of participants with AHI reductions at week 26 of  ≥30% to  ≥90%, by 10% increments, is shown in Figure 3A. Greater AHI response was observed among participants in the AD109 arm relative to placebo at each decile. Reductions  ≥70% in AHI at week 26 were reported in 17.2% (52/302) of the AD109 arm vs 8.9% (28/313) in the placebo arm (*P* = .003; Figure 3A). Similar AD109 treatment effects were observed in the supportive on-treatment estimand (Figure 2F, Figure 3B, Figure S7).

To understand the nature of the AD109 benefits on AHI, further analyses were conducted on stage- and position-specific metrics. AD109 reduced the AHI during both non-REM and REM sleep stages, with reductions reaching statistical significance in the non-REM stages (Table 4). AHI was also significantly reduced in the supine position, suggesting that the effect of AD109 is independent from differences in time spent supine on treatment vs baseline (Table 4). Additional data on arousal indices are provided in Table 5. AD109 reduced snoring vs placebo (Table S2).

**Table 4:** Change in non-REM AHI, REM AHI, and supine AHI at week 26 (exploratory endpoints; on-treatment estimand)^a^.

**On-treatment estimand (*N*** = **556)**						
**Non-REM AHI**						
**Median (IQR), events/h [*n*]**				**Change from baseline to week 26, LS mean (95% CI), events/h**		**Estimated treatment difference of LS means [AD109 − placebo] (95% CI) and *P* value**
**AD109 (*n* = 260)**		**Placebo (*n*** = **296)**		**AD109**	**Placebo**	
**Baseline**	**Week 26**	**Baseline**	**Week 26**			
**17.4 (10.2-26.8) [260]**	10.7 (4.4-23.1) [234]	16.7 (10.1-26.8) [296]	13.4 (6.0-28.4) [269]	−3.7 (−5.6 to −1.8)	0.8 (−1.0 to 2.6)	−4.5^b^ (−7.1 to −2.0)
						*P* <.001
**REM AHI, excluding participants with <10 minutes of REM sleep**						
**Median (IQR), events/h [*n*]**				**Change from baseline to week 26, LS mean (95% CI), events/h**		**Estimated treatment difference of LS means [AD109 − placebo] (95% CI) and *P* value**
**AD109 (*n* = 260)**		**Placebo (*n*** = **296)**		**AD109**	**Placebo**	
**Baseline**	**Week 26**	**Baseline**	**Week 26**			
**30.5 (15.5-47.2) [249]**	28.2 (11.6-46.9) [157]	30.9 (14.9-47.7) [289]	30.8 (12.8-49.2) [257]	−3.6 (−6.7 to −0.5)	−0.2 (−2.7 to 2.3)	−3.4^c^ (−7.4 to 0.6)
						*P* = .092
**Supine AHI, excluding participants with <10 minutes of supine sleep**						
**Median (IQR), events/h [*n*]**				**Change from baseline to week 26, LS mean (95% CI), events/h**		**Estimated treatment difference of LS means [AD109 − placebo] (95% CI) and *P* value**
**AD109 (*n* = 260)**		**Placebo (*n*** = **296)**		**AD109**	**Placebo**	
**Baseline**	**Week 26**	**Baseline**	**Week 26**			
**29.0 (18.4-42.2) [253]**	18.7 (9.1-38.6) [228]	28.2 (18.6-44.5) [292]	24.7 (11.4-43.0) [259]	−7.4 (−10.0 to −4.8)	−2.5 (−4.9 to −0.1)	−4.9^d^ (−8.4 to −1.4)
						*P* = .006

Abbreviations: AHI, apnea–hypopnea index based on 4% hypopnea desaturation; CI, confidence interval; IQR, interquartile range; LS, least squares; REM, rapid eye movement.

a Analysis was prespecified for on-treatment estimand.

b The placebo-adjusted reduction at week 4 was −5.5 (−7.5 to −3.5); *P* <.0001.

c The placebo-adjusted reduction at week 4 was −6.4 (−9.7 to −3.1); *P* <.001.

d The placebo-adjusted reduction at week 4 was −8.1 (−11.1 to −5.1); *P* <.0001.

**Table 5:** Arousal indexes at week 26 (exploratory endpoints; on-treatment estimand)^a^.

**On-treatment estimand (*N*** = **556)**					
**Arousal index—respiratory**					
**Median (IQR), events/h [*n*]**				**Change from baseline to week 26, median (IQR), events/h**	
**AD109 (*n* = 260)**		**Placebo (*n*** = **296)**		**AD109 (*n*** = **260)**	**Placebo (*n*** = **296)**
**Baseline**	**Week 26**	**Baseline**	**Week 26**	0.1 (−5.0 to 5.8)	1.3 (−3.5 to 7.5)
**12.5 (8.2-19.5) [260]**	13.4 (7.2-21.5) [234]	13.0 (8.7-20.2) [296]	15.3 (8.4-22.2)[269]		
**Arousal index—total**					
**Median (IQR), events/h [*n*]**				**Change from baseline to week 26, median (IQR), events/h**	
**AD109 (*n* = 260)**		**Placebo (*n*** = **296)**		**AD109 (*n*** = **260)**	**Placebo (*n*** = **296)**
**Baseline**	**Week 26**	**Baseline**	**Week 26**	2.9 (−2.5 to 10.5)	1.6 (−3.9 to 8.7)
**17.2 (12.1-24.5) [260]**	21.6 (14.2-31.5) [234]	17.6 (12.3-24.8) [296]	20.0 (12.8-27.8) [269]		

Abbreviation: IQR, interquartile range.

a Analysis was prespecified for on-treatment estimand.

The evaluation of the treatment effect on AHI and HB at week 26 performed with an adjusted model including the change from baseline in proportion of REM sleep and BMI is provided in Table S3 and shows that the reduction in AHI is independent from reduction in REM and BMI in the treatment arm.

Predicted absolute changes in AHI from model estimates at week 26 are shown in Figure S8. Additional analyses of AHI, ODI, and HB in the subset of participants with completed assessments at week 4 and at week 26 are provided in Figure S9.

### Safety

AEs were reported by 70.8% (230/325) of the participants who received AD109 and 46.7% (149/319) of those who received placebo (Table 6). The most common (occurring in  ≥10% of participants) AEs in the AD109 arm were dry mouth, insomnia, and nausea. The most common AEs were generally mild in severity (Table S4).

**Table 6:** Summary of overall safety (safety analysis set).

	**AD109^a^ (*n*** = **325)**	**Placebo (*n*** = **319)**
**Overview of TEAEs^b^, *n* (%)**		
**Any TEAE**	230 (70.8)	149 (46.7)
**Any treatment-related^c^ TEAE**	190 (58.5)	75 (23.5)
**Any severe^d^ TEAE**	15 (4.6)	10 (3.1)
**Any serious^e^ TEAE**	5 (1.5)^f^	8 (2.5)^g^
**Any TEAE associated with fatal outcome**	0	0
**Any TEAE leading to treatment discontinuation**	69 (21.2)	10 (3.1)
**Most common TEAEs (occurring in ≥5% of participants in either arm), *n* (%)**		
**Dry mouth**	115 (35.4)	29 (9.1)
**Insomnia^h^**	67 (20.6)	15 (4.7)
**Nausea**	37 (11.4)	2 (0.6)
**Urinary hesitation**	29 (8.9)	1 (0.3)
**Constipation**	17 (5.2)	4 (1.3)
**Somnolence**	17 (5.2)	10 (3.1)
**Primary TEAEs^c^ leading to treatment discontinuation (occurring in ≥2 participants in either arm), *n* (%)**		
**Insomnia^h^**	16 (4.9)	0
**Urinary hesitation**	8 (2.5)	0
**Nausea**	7 (2.2)	0
**Erectile dysfunction/sexual dysfunction**	5 (1.5)	0
**Dry mouth**	4 (1.2)	0
**Hypertension**	3 (0.9)	0
**Palpitations**	3 (0.9)	0
**Abdominal pain, upper**	2 (0.6)	0
**Feeling jittery**	2 (0.6)	0
**Urinary retention**	2 (0.6)	0

Abbreviation: TEAE, treatment-emergent adverse event.

a Two participants who received AD109 were assigned to placebo at randomization.

b Adverse events were coded by Medical Dictionary for Regulatory Activities version 27.0 or higher. A TEAE is defined as an adverse event with an onset or diagnosis after the first dose of study medication and on or before the end of treatment (last dose of study medication + 7 days), regardless of causality.

c Based on investigator assessment as related, possibly related or probably related; an adverse event is considered treatment-related if the relationship attribution designation is missing.

d An adverse event with missing intensity is considered as severe.

e An adverse event is considered serious if missing seriousness status.

f In the AD109 arm, 5 participants experienced the following serious adverse events (all unrelated to AD109 treatment): 1 participant had cholelithiasis and cholestatic hepatitis, 1 participant had gastritis and bacterial pneumonia, 1 participant had asthma and chronic obstructive pulmonary disease, 1 participant had cholecystitis, and 1 participant had malignant melanoma.

g In the placebo arm, 8 participants experienced the following serious adverse events (all unrelated to placebo treatment): 1 participant had road traffic accident, sternal fracture, thoracic vertebral fracture, urinary bladder rupture, and rib fracture, 1 participant had respiratory failure and metabolic encephalopathy, 1 participant had cholelithiasis, 1 participant had bile duct stone, 1 participant had acute pancreatitis, 1 participant had perforated appendicitis, 1 participant had influenzal pneumonia and transient ischemic attack, and 1 participant had asthma.

h Includes initial insomnia, insomnia, and middle insomnia.

Treatment-related AEs are provided in Table S5. Serious AEs were reported by 1.5% (5/325) of participants who received AD109 and 2.5% (8/319) of participants who received placebo; all were deemed by investigators to be unrelated to study medication (Table 6).

Overall, 21.2% (69/325) of participants receiving AD109 and 3.1% (10/319) of participants receiving placebo discontinued treatment due to an AE (Table 6). AE-related discontinuations occurred predominantly early after treatment initiation, with markedly fewer events later in the treatment period (Figure S10). The primary (occurring in  ≥7 participants) AEs leading to AD109 discontinuation were insomnia, urinary hesitation, and nausea (Table 6). Additional data on sleep architecture are provided in Table 7. No deaths were reported in the trial. Changes in BMI at week 26 were small across treatment groups; median change was −0.1 kg/m^2^ (IQR, −1.1 to 0.6) with AD109 and 0.1 kg/m^2^ (IQR, −0.4 to 0.9) with placebo. Mean changes in heart rate and blood pressure from baseline to week 26 were small (Table 8), and the proportions of participants with elevated heart rate and blood pressure across treatment arms are shown in Table 8.

**Table 7:** Total sleep time and percentage of sleep efficiency and sleep stages^a^ at week 26 (exploratory endpoints; safety analysis set)^b^.

**Safety analysis set (*N*** = **644)**					
**Total sleep time**					
**Median (IQR), min [*n*]**				**Change from baseline to week 26, median (IQR), min**	
**AD109 (*n* = 325)**		**Placebo (*n*** = **319)**		**AD109 (*n*** = **270)**	**Placebo (*n*** = **275)**
**Baseline**	**Week 26**	**Baseline**	**Week 26**	−8.8 (−52.1 to 35.0)	7.5 (−27.2 to 40.0)
**377.5 (329.9-413.0) [325]**	374.3 (319.0-409.1) [270]	379.5 (335.0-412.8) [319]	392.5 (340.0-426.1) [275]		
**Sleep efficiency**					
**Median (IQR), % [*n*]**				**Change from baseline to week 26, median (IQR), %**	
**AD109 (*n* = 325)**		**Placebo (*n*** = **319)**		**AD109 (*n*** = **270)**	**Placebo (*n*** = **275)**
**Baseline**	**Week 26**	**Baseline**	**Week 26**	−1.9 (−10.3 to 7.8)	0.6 (−5.9 to 8.1)
**78.1 (68.6-85.7) [325]**	78.0 (66.5-85.2) [270]	79.5 (70.5-85.9) [319]	81.8 (72.2-88.2) [275]		
**Stage N1**					
**Median (IQR), % [*n*]**				**Change from baseline to week 26, median (IQR), %**	
**AD109 (*n* = 325)**		**Placebo (*n*** = **319)**		**AD109 (*n*** = **270)**	**Placebo (*n*** = **275)**
**Baseline**	**Week 26**	**Baseline**	**Week 26**	3.0 (−1.7 to 7.8)	−0.3 (−3.8 to 3.8)
**12.2 (7.9-18.0) [325]**	15.8 (9.9-23.0) [270]	12.7 (7.6-17.5) [319]	11.7 (7.6-17.8) [275]		
**Stage N2**					
**Median (IQR), % [*n*]**				**Change from baseline to week 26, median (IQR), %**	
**AD109 (*n* = 325)**		**Placebo (*n*** = **319)**		**AD109 (*n*** = **270)**	**Placebo (*n*** = **275)**
**Baseline**	**Week 26**	**Baseline**	**Week 26**	7.3 (−0.8 to 13.8)	2.2 (−4.4 to 10.0)
**60.8 (54.1-68.2) [325]**	66.9 (58.9-75.5) [270]	60.8 (53.7-66.6) [319]	62.9 (56.5-68.2) [275]		
**Stage N3**					
**Median (IQR), % [*n*]**				**Change from baseline to week 26, median (IQR), %**	
**AD109 (*n* = 325)**		**Placebo (*n*** = **319)**		**AD109 (*n*** = **270)**	**Placebo (*n*** = **275)**
**Baseline**	**Week 26**	**Baseline**	**Week 26**	−1.6 (−6.1 to 0.6)	−1.3 (−6.1 to 0.8)
**7.6 (2.1-15.1) [325]**	3.9 (0.1-10.2) [270]	7.9 (2.8-15.2) [319]	5.3 (0.7-13.1) [275]		
**Stage REM**					
**Median (IQR), % [*n*]**				**Change from baseline to week 26, median (IQR), %**	
**AD109 (*n* = 325)**		**Placebo (*n*** = **319)**		**AD109 (*n*** = **270)**	**Placebo (*n*** = **275)**
**Baseline**	**Week 26**	**Baseline**	**Week 26**	−7.5 (−13.6 to −0.9)	−0.2 (−5.0 to 5.2)
**15.6 (10.8-20.7) [325]**	7.2 (1.7-14.3) [270]	15.9 (11.6-21.0) [319]	16.9 (11.4-20.1) [275]		
**Supine sleep time**					
**Median (IQR), % [n]**				**Change from baseline to week 26, median (IQR), %**	
**AD109 (*n* = 325)**		**Placebo (*n*** = **319)**		**AD109 (*n*** = **270)**	**Placebo (*n*** = **275)**
**Baseline**	**Week 26**	**Baseline**	**Week 26**	0.3 (−14.9 to 14.9)	2.8 (−10.4 to 18.2)
**47.3 (32.5-61.6) [325]**	47.2 (30.1-65.0) [270]	45.2 (32.4-61.2) [319]	49.9 (33.7-67.0) [275]		

Abbreviations: IQR, interquartile range; REM, rapid eye movement; SD, standard deviation.

a Sleep stages according to the American Academy of Sleep Medicine scoring manual version 3.^60^

b This analysis on the safety set was post hoc.

**Table 8:** Vital signs of heart rate and blood pressure and proportion with high heart or blood pressure (safety analysis set).

Vital signs of heart rate and blood pressure at baseline and at week 26						
	Heart rate (beats/min)		Systolic blood pressure (mm Hg)		Diastolic blood pressure (mm Hg)	
	**AD109 (*n*** = **325)**	**Placebo (*n*** = **319)**	**AD109 (*n*** = **325)**	**Placebo (*n*** = **319)**	**AD109 (*n*** = **325)**	**Placebo (*n*** = **319)**
**Baseline, mean (SD) [*n*]**						
** Day 1 (evening before PSG)**	75.9 (10.1) [324]	76.3 (10.9) [318]	124.2 (11.7) [324]	125.6 (11.6) [318]	78.5 (7.9) [324]	79.4 (8.0) [318]
** Day 2 (morning after PSG)**	70.5 (9.6) [323]	70.4 (9.2) [318]	124.9 (11.5) [324]	126.1 (11.5) [318]	80.3 (7.9) [324]	80.3 (7.8) [318]
**Week 26, mean (SD) [*n*]**						
** Day 1 (evening before PSG)**	77.5 (11.2) [268]	74.2 (10.6) [278]	126.0 (10.9) [268]	127.0 (12.4) [278]	79.4 (6.8) [268]	78.9 (7.5) [278]
** Day 2 (morning after PSG)**	74.1 (9.6) [269]	70.4 (10.4) [276]	125.3 (11.4) [269]	126.1 (12.0) [276]	80.5 (8.0) [269]	79.4 (7.7) [276]
**Mean change from baseline to week 26 (SD) [*n*]**						
** From baseline day 1 to week 26 day 1**	1.5 (9.8) [268]	−1.9 (10.1) [277]	1.4 (11.8) [268]	1.7 (11.9) [277]	0.9 (8.0) [268]	−0.5 (7.7) [277]
** From baseline day 2 to week 26 day 2**	3.6 (9.4) [267]	0.0 (9.2) [276]	−0.2 (11.5) [268]	0.1 (11.7) [276]	−0.2 (8.2) [268]	−0.7 (8.1) [276]

Proportion of participants with high heart rate and/or high blood pressure at baseline and any time on treatment				
		**AD109 (*n*** = **325)**	**Placebo (*n*** = **319)**	*P* value
**Heart rate, *n* (%) [*n*]**				
**≥100 beats/min**	**Baseline**	7 (2.2)	6 (1.9)	.805
	**On study**	20 (6.3)	6 (1.9)	.006
		[316]	[309]	
**Systolic blood pressure, *n* (%) [*n*]**				
**≥140 mm Hg**	**Baseline**	40 (12.3)	48 (15.0)	.312
	**On study**	83 (26.3)	82 (26.5)	.939
		[316]	[309]	
**Diastolic blood pressure, *n* (%) [*n*]**				
**≥90 mm Hg**	**Baseline**	40 (12.3)	47 (14.7)	.368
	**On study**	65 (20.6)	64 (20.7)	.956
		[316]	[309]	

Abbreviations: PSG, polysomnography; SD, standard deviation.

## Discussion

In participants with mild-to-severe OSA who had failed or refused PAP therapy, treatment with AD109 resulted in statistically significant improvements in objective measures of disease severity and oxygenation, consistent with the phase 2b MARIPOSA trial.^31^ AD109 demonstrated multiple benefits over varying domains of physiologic, symptomatic, and categorical endpoints that we believe support clinical meaningfulness of AD109 treatment in patients with OSA.

Approximately 1 in 5 participants discontinued AD109 due to AEs. The differences in effect sizes observed in the ITT vs on-treatment estimand were likely caused by the relatively high discontinuation rate. The population studied must have been intolerant to or refused PAP therapy to enroll; thus, the trial may have been enriched with participants who may have exhibited some degree of subclinical insomnia.^41^ However, insomnia was not assessed in a standardized manner in this trial. Future studies will need to assess whether standardized screening for insomnia can better identify patients who will not tolerate AD109 well. For many patients, a short-term trial may be the most straightforward approach to assess tolerability given the mild and reversible adverse effects tending to occur early after treatment initiation.

In addition, participants who experienced a higher incidence of AEs may have benefited from a lower, efficacious dose of AD109.^30^ However, dose reduction was not allowed in the trial. Notably, the high proportion of discontinuations due to AEs occurred predominantly early after treatment initiation. In contrast, non-AE-related discontinuations were similar between the AD109 and placebo arms and were distributed more uniformly over time and generally at lower counts. Overall, these findings suggest that tolerability-driven discontinuation tended to occur early after treatment initiation.

In the ITT analysis, AD109 significantly reduced AHI from baseline to week 26 by a model-estimated 44.1%; in the on-treatment estimand, we observed a 55.6% reduction in AHI. At the group level, treatment was associated with a shift in disease severity from the moderate to mild range (from a median of 19.8 at baseline to 13.3 at week 26 in the ITT, and from a median of 19.3 at baseline to 10.6 at week 26 in the on-treatment estimand). This magnitude of improvement, we believe, is clinically meaningful, as mild OSA has been associated with lower cardiovascular, metabolic, and neurocognitive risk compared to moderate OSA.^42-44^ At 26 weeks, >25% of participants in the AD109 arm achieved  ≥50% reduction in AHI, a clinically meaningful improvement based on American Academy of Sleep Medicine guidelines,^45^^,^^46^ although this effect was not statistically significant compared with the placebo arm. Clinically meaningful response was observed as early as week 4 based on the ≥50% reduction in AHI responder rates, suggesting that the lower treatment difference at week 26 could be attributed to the higher early treatment discontinuation rate with AD109 relative to placebo, since participants who discontinued treatment were classified as nonresponders in this analysis.

In contrast, in analyses using alternative responder definitions (≥30% to  ≥90% AHI reduction, in 10% increments), participants who discontinued treatment were retained in the analysis, and the AD109 arm demonstrated consistently higher response rates than placebo across all thresholds in the ITT set. Furthermore, although AD109 was associated with small reductions in weight and proportion of REM sleep, our adjusted analyses found that these changes had minimal influence on the improvements in AHI observed, supporting that the proposed mechanism of action of AD109 is via improvements in sleep-related neuromuscular dysfunction.

Treatment with AD109 enabled 17.6% of the participants to achieve complete OSA disease control (AHI <5 events/hour) at 26 weeks. In supportive analyses, AD109 improved AHI across diverse subgroups, such as those defined by sex, weight class, and baseline OSA severity (mild, moderate, and severe), supporting the generalizability of study findings to a broad spectrum of patients with OSA.

Participants who received AD109 also experienced significant improvements in oxygenation, evidenced by reduction in ODI at 26 weeks. AD109 was associated with improvements in HB, although formal statistical significance was not achieved under the prespecified hierarchical testing. As HB captures frequency, depth, and duration of oxygen desaturations and has been linked to increased risk of cardiovascular sequelae and early mortality, these improvements suggest the possibility of clinically meaningful reductions in chronic intermittent hypoxia.^47^^,^^48^

Patients with OSA exhibit a broad spectrum of symptoms, with some individuals experiencing fatigue and excessive daytime sleepiness, whereas many others (up to 60%) report no OSA-related symptoms.^49^^,^^50^ Fatigue and excessive daytime sleepiness can negatively impact the quality of life of patients with OSA, although the presence and severity of symptoms do not correlate with measures of objective disease severity.^51^ The substantial improvement in symptoms observed in both arms suggests that the placebo effect may have impaired the ability to detect true clinical improvements. Although we did not observe a greater improvement in fatigue overall with AD109 compared to placebo, in participants with more daytime symptoms at baseline, AD109 led to improvements in symptoms of fatigue relative to placebo.

AD109 demonstrated a manageable safety profile for the majority of participants who remained on therapy, with predominantly mild AEs exhibited in participants with mild-to-severe OSA. The most commonly observed AEs were dry mouth, insomnia, and nausea, and consistent with earlier AD109 clinical trials and with the known AE profiles of the constituent compounds of AD109. Small changes in sleep architecture were observed consistent with the higher prevalence of insomnia. A higher percentage of participants experienced AEs leading to permanent discontinuation of study medication with AD109 versus the placebo group (21.2% vs 3.1%). Changes in blood pressure and heart rate were small, although a small subgroup of participants showed tachycardia while on AD109. No serious AEs related to AD109 were reported.

A very large unmet medical need remains for patients with OSA who remain untreated or undertreated or discontinue treatment with currently available therapies.^18^^,^^52^ The current standard of care for OSA treatment has various limitations, with most patients lacking effective treatment options.^28^^,^^46^^,^^53^^,^^54^ It is estimated almost 50% of patients discontinue PAP during the course of 1 year.^20^ Recent evidence suggests that patients with OSA desire an oral medication option.^55^

AD109 is an investigational, once-daily, fixed-dose, oral combination of the novel antimuscarinic aroxybutynin (2.5 mg) and the selective norepinephrine reuptake inhibitor atomoxetine (75 mg), in one tablet.^30-33^ Treatment with AD109 provides clinical improvements to participants with OSA who cannot accept or adhere to PAP therapy, with a 44.1% reduction in AHI seen at 26 weeks vs placebo. As early as week 4, the SynAIRgy trial demonstrated highly statistically significant (*P* <.001) and clinically responsive effects, consistent with an early onset of action for AD109. In the SynAIRgy trial, AD109 was studied across a wide spectrum of participants with mild-to-severe OSA across a varied range of weight classes (BMI 18.5-42 kg/m^2^) and levels of disease severity. Because the proposed mechanism of action addresses the sleep-related neuromuscular dysfunction, a root cause of OSA affecting all patients, AD109 has the potential to benefit a wider population than other current treatment options.^28^^,^^54^ As an oral once-daily-at-bedtime treatment, AD109 may offer a simpler, more acceptable alternative for the large number of patients with diagnosed but untreated OSA. The strengths of the SynAIRgy trial include its trial size of >600 participants, sex balance, and diverse racial and ethnic participant enrollment, across all severity classes of OSA and across a wide range of weight classes, including approximately one-third of the study population that was not obese.

Limitations of this trial include that the substantial drug discontinuation rate before 26 weeks (34.3% for AD109 vs 16.3% for placebo) likely diminished the magnitude of benefits observed. The greater effects of AD109 observed in the on-treatment estimand analyses and the greater benefits observed in endpoints at week 4, when discontinuation rates were lower, support this interpretation. Dose reduction was not allowed in this trial; however, it could have resulted in improved tolerability. The population studied skewed toward mild-to-moderate baseline disease severity, which can contribute to limited absolute effects despite meaningful proportional improvements. Nonetheless, modest changes can translate into improvements in physiological parameters and cardiovascular protection,^56-59^ and we observed on AD109, at the group level, a shift from the moderate to mild OSA severity category. In the context of PRO assessments, participants were never specifically asked if they were aware of receiving placebo, so we cannot rule out the possibility of expectation bias influencing responses. Additionally, trial sites were limited to the United States and Canada and may not fully represent OSA in other geographies.

Results from the SynAIRgy trial indicate that AD109, an investigational first-in-class oral drug, meets statistically significant and clinically meaningful endpoints for the treatment of mild-to-severe OSA. If approved, AD109 may be a treatment option for patients who do not tolerate or refuse PAP therapy, with utility across all levels of disease severity and a wide range of weight classes (BMI 18.5-42 kg/m^2^).

## Supplementary Material

aamag215_Supplementary_Data

## Data Availability

The datasets generated and/or analyzed in the present study (including de-identified individual participant data, data dictionary, and study documents) will be shared via secure email or secure website with a signed data access agreement to scientifically qualified investigators who provide a methodologically sound proposal that is aligned with company’s areas of scientific interest and reviewed/approved by committee. This article has an online data supplement, which is accessible from the Supplements tab.
