# Supplementary material for: Aroxybutynin and atomoxetine (AD109) for obstructive sleep apnea: a randomized phase 3 trial (SynAIRgy)
Source: Am J Respir Crit Care Med. 2026 May 18;212(7):1569–84. doi: 10.1093/ajrccm/aamag215 (PMC13318230; doi:10.1093/ajrccm/aamag215)
Supplement: aamag215_Supplementary_Data [file aamag215_supplementary_data.zip › Strollo_SynAIRgy_supplement_clean.docx]

Aroxybutynin and Atomoxetine (AD109) for Obstructive Sleep Apnea: A Randomized Phase 3 Trial (SynAIRgy)

Patrick J. Strollo Jr, Ron Farkas, Luigi Taranto-Montemurro, John Cronin, and Sanjay R. Patel; on behalf of SynAIRgy Investigators

# Online Data Supplement

# List of SynAIRgy Investigators

# Supplementary Methods

1. Study Inclusion and Exclusion Criteria
2. Guidelines for Administration of Concomitant Medication
3. Sample Size and Statistical Power
4. Randomization and Blinding
5. Key Secondary Patient-Reported Endpoints
6. Additional Secondary Endpoints
7. Statistical Analysis of the Primary Endpoint and the Secondary Endpoints
8. Subgroup Analyses of the Primary Endpoint and the Secondary Endpoints
9. Statistical Analysis of Exploratory Endpoints
10. Safety Assessments
11. Post Hoc Analyses
12. Changes in the Conduct of the Study

# Supplementary Figures

**Figure E1.** SynAIRgy study design.

**Figure E2.** Baseline distribution of AHI (Intent to Treat)

**Figure E3.** Subgroup analyses of the mean change in AHI from baseline to week 26 (primary endpoint) by intent to treat (*A*) and on-treatment estimands (*B*).

**Figure E4.** Mean percent reduction in HB from baseline to weeks 4 and 26 (secondary key efficacy endpoint) by intent to treat (*A*) and on-treatment estimands (*B*).

**Figure E5.** Subgroup analyses of the fold change in HB from baseline to week 26 (secondary key efficacy endpoint) by intent to treat (*A*) and on-treatment estimands (*B*).

**Figure E6.** Select subgroup analyses of the mean change in PROMIS-Fatigue T-score from baseline to week 26 (secondary key efficacy endpoint) by intent to treat (*A*) and on-treatment estimands (*B*).

**Figure E7.** Proportion of participants with change in OSA severity category from baseline to week 26 (exploratory endpoint; on-treatment estimand).

**Figure E8.** Model-estimated change from baseline in AHI at Week 26 (Intent to Treat and On-Treatment Estimand)

**Figure E9.** Mean change from baseline to Weeks 4 and 26 in AHI (*A*), ODI (*B*), and HB (C) in the on-treatment estimand.

**Figure E10.** Timing of study discontinuations in the AD109 arm (safety analysis set).

**Supplementary Tables**

**Table E1.** Observed Values for Primary and Key Secondary Endpoints at Baseline and at Week 26 (Intent to Treat and On-Treatment Estimand)

**Table E2.** Snoring Analysis (Exploratory Endpoint; On-Treatment Estimand)

**Table E3.** Treatment effect on AHI and HB at Week 26 Performed with Adjusted Model Including the Change from Baseline in Proportion of REM Sleep (On-Treatment Estimand)

**Table E4.** Summary of Adverse Events Occurring in ≥5% of Participants in Either Arm, by Severity (Safety Analysis Set)

**Table E5.** Summary of Adverse Events Occurring in ≥5% of Participants in Either Arm, by Relationship (Safety Analysis Set)

**List of SynAIRgy Investigators (Study NCT05813275/APC-APN-305):**

| **Principal Investigator** | **Clinical Trial Site** | **Location** | **Country** |
| --- | --- | --- | --- |
| Akinyemi Ajayi | Florida Pediatric Research Institute | Winter Park, FL | USA |
| Bernadette Alejandrino | Providere Research Inc | West Covina, CA | USA |
| Jerome Alonso | Canadian Sleep Consultants | Calgary, AB | Canada |
| Najib Ayas | Vancouver Coastal Health Research Institute | Vancouver, BC | Canada |
| Francisco Badar | Core Healthcare Group | Cerritos, CA | USA |
| Jacob Coleman | Javara Inc - Tryon Medical Partners PLCC | Charlotte, NC | USA |
| William Cooper | Javara Inc-Privia Medical Group | Thomasville, GA | USA |
| Bruce Corser | Sleep Management Institute (Intrepid Research) | Cincinnati, OH | USA |
| Ronald Cridland | Medical Arts Health Research | Kelowna, BC | Canada |
| Dominick D'Aunno | The Heights Hospital (HD Research) | Houston, TX | USA |
| Matthew Davis | Neurology Specialists of Monmouth County | West Long Branch, NJ | USA |
| Bertrand De Silva | Probate Clinical Research Corporation | Riverside, CA | USA |
| Michael Downing | FutureSearch Trials of Dallas LP | Dallas, TX | USA |
| Alaa El-Gendy | Florida Lung and Sleep Associates | Lehigh Acres, FL | USA |
| Tomas Fiel | Fiel Family Sports Medicine - PC CCT Research | Tempe, AZ | USA |
| Steven Geller | Centennial Medical Group | Elkridge, MD | USA |
| James Geyer | Alabama Neurology & Sleep Medicine | Tuscaloosa, AL | USA |
| Andrew Gould | Advanced ENT and Allergy | Louisville, KY | USA |
| Nella Green | Exalt Clinical Research | Chula Vista, CA | USA |
| Mario Guillen | Canvas Clinical Research | Lake Worth, FL | USA |
| John Hemmersmeier | South Ogden Family Medicine CCT Research | South Ogden, UT | USA |
| John Hudson | FutureSearch Trials of Neurology | Austin, TX | USA |
| Monica Jaffe | Chicago Research Center | Chicago, IL | USA |
| Thomas Jarrett | Peters Medical Research, LLC | High Point, NC | USA |
| John Khoury | Abington Neurological Associates | Abington, PA | USA |
| John Kimoff | McGill University Health Centre | Montreal, QC | Canada |
| Oleg Kouskov | St. Lukes Clinic - Idaho Pulmonary Associates | Meridian, ID | USA |
| Michael Lacey | NeuroTrials Research | Atlanta, GA | USA |
| Judith Leech | West Ottawa Sleep Centre | Ottawa, ON | Canada |
| David Lesch | Georgia Neurology and Sleep Medicine Associates | Suwanee, GA | USA |
| Michael Lillestol | Lillestol Research LLC | Fargo, ND | USA |
| Reinero Linares - Mera | JSV Clinical Research Study Inc | Tampa, FL | USA |
| Alan Lowe | AMNDX | Markham, ON | Canada |
| Kinjal Madhav | Profound Research LLC (North County Neurology Associates) | Carlsbad, CA | USA |
| David Marks | Element Research Group | San Antonio, TX | USA |
| Ronald Mayfield | Tribe Clinical Research LLC | Greenville, SC | USA |
| James Maynard | CTI CRC | Cincinnati, OH | USA |
| Jessica McCoun | Atlanta Center for Medical Research LLC | Atlanta, GA | USA |
| Tatyana Miroshnikova | Clinical Neuroscience Solutions, Inc - Orlando | Orlando, FL | USA |
| Rizwana Mohseni | Catalina Research Institute | Montclair, CA | USA |
| Andrew Pastewski | Nouvelle Clinical Research | Cutler Bay, FL | USA |
| Paresh Patel | Lone Star Lung and Sleep Clinic | Houston, TX | USA |
| Sanjay Patel* | Division of Pulmonary, Allergy, Critical Care Medicine and Sleep Medicine, Department of Medicine, University of Pittsburgh | Pittsburgh, PA | USA |
| Susheel Patil | University Hospital of Cleveland Medical Center | Cleveland, OH | USA |
| Nirupa Paulraj | Las Vegas Clinical Trials | North Las Vegas, NV | USA |
| Enrique Pelayo | Advanced Medical Research Institute | Miami, FL | USA |
| Dena Petersen | Noble Clinical Research | Tucson, AZ | USA |
| Alec Platt | Respiratory Specialists | Wyomissing, PA | USA |
| Lew Pliamm | Canadian Phase Onward Inc. | Toronto, ON | Canada |
| Bruce Rankin | Accel Research Sites | Deland, FL | USA |
| Syed Raza | Revive Research Institute | Lathrup Village, MI | USA |
| Anne Romaker | UC Health Sleep Medicine | Cincinnati, OH | USA |
| Mark Rosenthal | Clinical Site Partners | Miami, FL | USA |
| Eugene Ryan | Chattanooga Research & Medicine PLLC | Chattanooga, TN | USA |
| Hector Sanchez | PMG Research of Wilmington | Wilmington, NC | USA |
| Andrew Schreiber | SDS Clinical Trials | Santa Ana, CA | USA |
| Sonja Schuetz | University of Michigan | Ann Arbor, MI | USA |
| Sudhir Sehgal | Huntsville Research Institute LLC | Huntsville, TX | USA |
| Colin Shapiro | Sleep and Alertness Center | Toronto, ON | Canada |
| Craig Shapiro | CenExel RCA - Hollywood | Hollywood, FL | USA |
| Gerald Shockey | Desert Clinical Research - CCT Research | Mesa, AZ | USA |
| Hermandeep Singh | Sleep Medicine Specialists of California (Tri-Valley Sleep Center) | San Ramon, CA | USA |
| Sushil Singhi | OnSite Clinical Solutions LLC/Carolina Cardiology Associates P.A. | Rock Hill, SC | USA |
| Steve Sitar | Orange County Research Institute | Anaheim, CA | USA |
| Eileen Sloan | Toronto Sleep Institute | Toronto, ON | Canada |
| Damien Stevens | The University of Kansas Medical Center | Kansas City, KS | USA |
| Kenneth Stiel | Foothills Research Center - CCT Research | Phoenix, AZ | USA |
| Patrick J. Strollo Jr.* | Veteran Affairs Pittsburgh Healthcare System, Pittsburgh, PA, USA and Division of Pulmonary, Allergy, Critical Care Medicine and Sleep Medicine, Department of Medicine, University of Pittsburgh | Pittsburgh, PA | USA |
| Masayoshi Takashima | Houston Methodist/Weill Cornell Medical College | Houston, TX | USA |
| Stephen Thein | Pacific Research Network | San Diego, CA | USA |
| Patrick Whitten | OSF HealthCare Saint Francis Medical Center | Peoria, IL | USA |
| Paul Wylie | Preferred Research Partners | Little Rock, AK | USA |
| Dragos Zanchi | Meris Clinical Research | Brandon, FL | USA |

* Investigator members of the steering committee.

# Supplementary Methods

1. **Study Inclusion and Exclusion Criteria**

*A. Inclusion Criteria*

- Age ≥18 years
- Apnea-hypopnea index (AHI) based on American Academy of Sleep Medicine (E1) criteria that requires 4% desaturation for hypopneas of 10–45 events/h
- ≤25% central or mixed apneas (as proportion of total apneas + hypopneas)
- Periodic limb movements arousal index ≤15
- Eight-item Patient-Reported Outcomes Measurement Information System (PROMIS)-Fatigue T-score ≥50.4
- Positive airway pressure (PAP) failure (defined as no PAP use for ≥3 months before randomization or return/removal of device from the home) or current PAP refusal (defined as refusal after prior positive sleep study or prior refusal of provider-recommended sleep study due to unwillingness to consider PAP)
- Body mass index (BMI) between 18.5 and 40 kg/m^2^ for men or between 18.5 and 42 kg/m^2^ for women
- Must have voluntarily agreed to participate in this study and sign an institutional review board–approved informed consent before any of the screening visit procedures
- Must have been able to understand the nature of the study and must have had the opportunity to have any questions answered

*B. Exclusion Criteria*

Medical Conditions

- Narcolepsy, restless legs syndrome requiring medication, rapid eye movement (REM) sleep behavior disorder
- Current bothersome symptoms of insomnia (difficulty initiating or maintaining sleep, as distinct from unrefreshing sleep or other symptoms attributable to obstructive sleep apnea [OSA]), as determined clinically by the site investigator at screening; the determination was made by use of clinical judgement rather than standardized insomnia instrument
  - Participants being treated for insomnia could have been enrolled only if the condition was stable and generally controlled in the investigator’s judgement
- Pierre Robin, Treacher Collins, or other craniofacial malformation syndrome, or grade ≥3 tonsillar hypertrophy
- Clinically significant or medically uncontrolled cardiovascular disease (e.g., ventricular arrhythmia requiring medical or device therapy), unstable atrial fibrillation (including cardioversion, ablation, or change in antiarrhythmic regimen within the prior 3 months [participants in whom anticoagulation was indicated must have been on such treatment for enrollment], or resting heart rate >100 beats/min), untreated or unstable coronary artery disease (including revascularization within 3 months), cardiac failure, cerebrovascular event, or transient ischemic attack or revascularization within 3 months
- Neuromuscular disorder (e.g., motor neuron disease, muscular dystrophy or myopathy, myasthenic syndrome); epilepsy; Parkinson’s disease, Alzheimer’s disease, or other neurodegenerative disease
- Schizophrenia, schizoaffective disorder, generalized anxiety disorder, or bipolar disorder according to *Diagnostic and Statistical Manual of Mental Disorders*, Fifth Edition (DSM-5) or International Classification of Diseases, 10th Revision criteria
- Attempted suicide within 1 year before screening, or current suicidal ideation
- Severe or frequent constipation considered currently bothersome by the participant, or symptomatic gastric motility disorder
- Current bothersome symptoms of bladder outlet obstruction, including difficulty initiating or maintaining urinary flow, straining, or sensation of incomplete bladder emptying; participants being treated with α-1 adrenergic antagonist for benign prostatic hypertrophy could have been enrolled only if the condition was stable and generally controlled
- Active substance use disorder as defined in DSM-5, or other substance use that in the investigator’s opinion would present an unreasonable risk to the participant or would interfere with their participation in the study or confound study interpretation
- A serious illness or infection 30 days before screening as determined by investigator
- Clinically significant cognitive dysfunction as determined by investigator
- Narrow-angle glaucoma
- Pregnant or nursing women

Prior/Concomitant Therapy

- PAP could have been initiated during the study if considered by the primary care provider and participant to have been in the participant’s best interest
- Participants with prior use of mandibular advancement devices, nasal devices, or sleeping position devices for the treatment of OSA could have enrolled as long as the devices were not used during participation in the study
- Participants with history of chronic oxygen therapy were excluded
- Participants with implanted devices for the treatment of OSA were excluded (e.g., hypoglossal nerve stimulator, or investigational devices)
- Participants taking drugs when used primarily or exclusively for weight loss were excluded (e.g., bupropion-naltrexone, liraglutide, semaglutide, tirzepatide orlistat, phentermine-topiramate)

Prior/Concurrent Clinical Study Experience

- Use of another investigational agent within 30 days or 5 half-lives, whichever was longer, before dosing

Diagnostic Assessments

- Montgomery-Åsberg Depression Rating Scale suicidal ideation response of thinking of or planning suicide (score ≥2)
- Systolic blood pressure up to 140 mm Hg or diastolic blood pressure up to 90 mm Hg was allowed in participants not currently treated with antihypertensives
- Unexplained or new identification of hepatic transaminases (aspartate transaminase or alanine transaminase) >2× the upper limit of normal (ULN) was generally exclusionary. However, elevation from 2 to 2.5 could be allowed with prior consent of the sponsor if evidence suggested it was due to nonalcoholic fatty liver disease or other condition that would not have been expected to acutely worsen or require medical intervention during the course of the study
- Total bilirubin >1.5× ULN (unless confirmed Gilbert syndrome)
- Estimated glomerular filtration rate <50 mL/min
- Electrocardiogram abnormality indicative of clinically significant cardiac disease that could be adversely affected by AD109

1. **Guidelines for Administration of Concomitant Medications**

Several concomitant medications were generally disallowed; however, if the medication was used at a sufficiently low dose, it may have been permitted with prior approval of the sponsor. A period of 1 month between discontinuing a prohibited medication and screening had to pass to allow determination that the participant’s medical condition was stable, but this period may have been shortened if appropriate in the clinical judgment of the investigator and with prior approval of the sponsor. On a case-by-case basis with approval of the sponsor, a medication that was typically disallowed could have been used to treat an intercurrent illness or condition that arose during the course of the study.

1. **Sample Size and Statistical Power**

The sample size was selected to achieve ≥90% power to detect a difference of at least 4 events/h in the apnea-hypopnea index based on 4% hypopnea desaturation (AHI) and 2.5 points on the PROMIS-Fatigue T-score, as these differences were considered clinically relevant for the planned population. In the previously conducted Phase 2 MARIPOSA trial (E2), the standard deviation of the placebo group change from baseline in AHI was approximately 9.0 events/h; for PROMIS-Fatigue T-score, it was 8.6 points. Based on the observed dropout rates in MARIPOSA (E2), it was assumed that the dropout rate in the placebo group would be 5% and the dropout rate in the AD109 group would be 25%. For the primary analysis, missing data were to be handled under a missing-not-at-random (MNAR) assumption by using multiple imputation from the control group. Based on the assumed standard deviation, anticipated dropout rates, and planned method of handling missing data, the required sample size was estimated using simulation. A total of 640 participants (320/treatment group) was expected to provide >98% power to detect a treatment difference of ≥4 events/h between treatment groups for the change from baseline to week 26 in AHI and ~90% power to detect a treatment difference of 2.5 points between treatment groups for the change in PROMIS-Fatigue T-score from baseline to week 26.

1. **Randomization and Blinding**

Randomization was centralized and conducted using interactive response technology. Each participant was assigned a unique number (randomization number) that encoded the participant’s assignment to one of the study arms, according to the randomization schedule. Study participants and their care providers were blinded to treatment assignment. Unblinding of a participant’s study treatment assignment was allowed if determined by the investigator to be necessary to protect participant safety.

1. **Key Secondary Endpoint: Hypoxic Burden**

The sleep apnea-specific Hypoxic Burden (HB) was evaluated as previously described (E3). Briefly, HB is a measure to capture the total amount of respiratory event-related hypoxemia over the sleep period. The HB was defined as the total area under the respiratory event-related desaturation curve divided by the total area of the sleep duration, with the units of HB being (%min)/h. HB was analyzed on the natural log scale of HB, i.e. log(HB) and results were back transformed. If HB was reported to be 0, log(0.01) was used in place of log(HB).

1. **Key Secondary Patient-Reported Endpoints**

PROMIS-Fatigue and PROMIS-Sleep Impairment measures were developed with modern psychometric techniques, including item response theory to assess various self-reported aspects of sleep and daytime impairment. Short versions for PROMIS-Fatigue and PROMIS-Sleep Impairment 8a were used. Each questionnaire consists of 8 items, and items are based on 5-point scales, either frequency or intensity, with higher scores corresponding to more severe or worse symptoms. The total raw score is the sum of all items. The total raw scores were translated into T-scores, with a mean of 50 and a standard deviation (SD) of 10 using scoring tables for PROMIS-Fatigue and PROMIS-Sleep Impairment, respectively.

1. **Additional Secondary Endpoints s (not Provided in this Report)**

Additional secondary efficacy endpoints evaluated at week 26 were change from baseline in Patient Global Impression of Severity (PGI-S) and Patient Global Impression of Change (PGI-C) scores for fatigue and change from baseline in Epworth Sleepiness Scale (ESS) scores. PGI-S for fatigue and PGI-C for fatigue are each single-item scales of the patient’s global impression (i.e., overall impression of either severity of fatigue proximate to the time point measured [PGI-S] or change from pretreatment in fatigue [PGI-C]). ESS is a self-administered questionnaire with 8 questions. Participants were asked to rate, on a 4-point scale (0–3), their usual chances of dozing off or falling asleep while engaged in 8 different activities in recent times. The ESS score (the sum of 8-item scores, 0–3) can range from 0 to 24. The higher the ESS score, the higher that person’s average sleep propensity in daily life, or their daytime sleepiness. The questionnaire takes approximately 2 or 3 minutes to answer.

1. **Statistical Analysis of the Primary Endpoint and the Secondary Endpoints**

All analyses and summary outputs were generated using SAS® version 9.4 or higher. All SAS programs used to generate analytical results were developed and validated according to SYNH programming standards and SAS validation procedures.

1. *Primary Endpoint and Secondary Endpoints Defined on a Continuous Scale*

The primary analysis methodology for the primary endpoint and the secondary endpoints defined on a continuous scale used a restricted maximum likelihood–based mixed model for repeated measurements (MMRM) in combination with the Newton-Raphson algorithm. Analyses included treatment, AHI strata, time, and treatment by time interaction as fixed effects and baseline score as a covariate. An unstructured covariance matrix was used to model the within-participant error. If the model failed to converge with an unstructured covariance matrix, a compound symmetric covariance matrix was used. When structured variance-covariance matrix was used to enable the model to converge, the “sandwich” estimator of the variance-covariance matrix was employed. The Kenward-Roger approximation was used to estimate the denominator degrees of freedom. The analysis was implemented using the MIXED procedure in SAS. Least squares means and treatment difference in least squares means for weeks 4 and 26 were reported along with the corresponding 95% CIs and the *P* values for the treatment group comparisons. The primary comparison was between treatment groups at week 26. All data collected up to week 26, regardless of adherence to investigational intervention, and initiation of PAP were used in the analysis. Some missing data at week 26 were expected with unequal distribution between treatment groups based on our previous Phase 2 study (MARIPOSA) (E2); therefore, missing data were assumed to be MNAR, and the endpoint was analyzed using a pattern-mixture model using a control-based approach by means of sequential modeling with multiple imputation, as described by O’Kelly (E4).

For the primary endpoint, no adjustment for multiplicity was needed and a 2-sided 5% significance level was used. Adjustment for multiplicity of the key secondary endpoints was conducted through a closed testing approach, where each key secondary endpoint was tested in a prespecified order. If the primary endpoint was statistically significant, then the first key secondary endpoint was tested using a 2-sided 5% significance level. If the first key secondary endpoint was significant, the second key secondary endpoint was tested using a 2-sided 5% significance level, and so on, until an endpoint was found to be not significant (using a 2-sided 5% significance level) or all key secondary endpoints had been tested. No adjustment for multiplicity was conducted for the non-key secondary endpoints.

Supportive analyses of the primary endpoint and the secondary endpoints defined on a continuous scale were performed using the intent to treat (ITT) set, with all data collected after a participant discontinued treatment set to missing. Missing data were assumed to be missing at random (MAR) and data were analyzed using an MMRM as outlined above. Supportive analyses of the primary endpoint and the secondary endpoints defined on a continuous scale were performed using the on-treatment estimand, with all data collected after a participant discontinued treatment set to missing. In this analysis, missing data were imputed using the last observation carried forward (LOCF) approach and then data were analyzed using an MMRM as outlined above.

1. *Key Secondary Endpoint Not Defined on a Continuous Scale*

The primary analysis of the proportion of participants with ≥50% reduction in AHI at week 26 (key secondary endpoint not defined on a continuous scale) was based on the stratified Cochran-Mantel-Haenszel (CMH) procedure, controlling for randomization strata, and estimates of the adjusted risk difference between treatment groups, along with the 2-sided 95% CI, were calculated based on the Mantel-Haenszel stratum weights (E5) and the Sato variance estimator (E6). Participants who discontinued treatment were considered nonresponders. In case of missing data (i.e., a participant did not discontinue treatment, but the week 26 polysomnography [PSG] (E7) was not completed or was not valid), data were imputed using a multiple imputation approach under an MAR assumption.

Supportive analyses of the proportion of participants with ≥50% reduction in AHI at week 26 (key secondary endpoint not defined on a continuous scale) were performed using the on-treatment estimand. If participants discontinued treatment prior to week 26, their last observation while on treatment was carried forward and the analysis was conducted using the CMH statistical method.

1. **Subgroup Analyses of the Primary Endpoint and the Secondary Endpoints**

The purpose of these analyses was to verify consistency of the efficacy results across subgroups. The following predefined subgroup analyses were performed for the primary and secondary endpoints, based on the ITT:

- Age, years: <65, ≥65
- Sex: male, female
- Race: Asian, Black or African American, White, other
- Baseline AHI: <15, ≥15 to <30, ≥30
- Baseline PROMIS-Fatigue T-score: <58.5, ≥58.5
- Baseline BMI, kg/m^2^: <18.5, 18.5–24.9, 25–29.9, 30–34.9, 35–39.9, ≥40 (after rounding to 1 decimal place); <18.5, and 18.5–24.9 were combined for the purpose of subgroup analysis

1. *Primary Endpoint and Secondary Endpoints Defined on a Continuous Scale*

Subgroup analyses only included participants with known subgroup data, and participants missing subgroup information were excluded. Missing data were imputed using the same approach used for the primary analysis. An assessment of consistency was made by evaluating the least squares means difference and associated 95% CI of the difference across subgroups. These estimates were obtained by fitting the MMRM model but including fixed effect terms for the subgroup and subgroup by treatment by visit interaction in the model. The least squares means, and treatment difference in least squares means for weeks 4 and 26, were reported along with the corresponding 95% CIs for each subgroup.

1. *Key Secondary Endpoint Not Defined on a Continuous Scale*

Subgroup analysis of the proportion of participants with ≥50% reduction in AHI at week 26 (key secondary endpoint not defined on a continuous scale) was performed using the ITT estimand. An assessment of consistency was made using a stratified CMH test, with subgroup as strata. The risk difference and associated 2-sided 95% CI (based on the Wald test) were reported for each subgroup. Missing data for subgroup inclusion were maintained as missing, and these participants were not included. Participants who discontinued treatment were considered nonresponders. Missing responses were imputed using the same approach used for the primary analysis (outlined in section 7B). The *P* value from the Breslow-Day test of homogeneity was reported (E8).

1. **Statistical Analysis of Exploratory Endpoints**
2. *Proportion of Participants with AHI Reduction of ≥30% to ≥90% by 10% Increments at Weeks 4 and 26*

Treatment groups were compared at each postbaseline time point using a stratified CMH test, controlling for randomization strata, with no adjustment for multiplicity. For the ITT estimand, participants with missing data for any reason were treated as a nonresponder. For the on-treatment estimand, the LOCF approach was used for missing data for any reason. The adjusted risk difference of the response rates between treatment groups along with the 2-sided 95% CI for the risk difference were calculated using the Mantel-Haenszel estimate based on the Mantel-Haenszel stratum weights (E5) and the Sato variance estimator (E6).

1. *Change From Baseline to Weeks 4 and 26 in OSA Severity Category (None, Mild, Moderate, and Severe)*

Treatment groups were compared using CMH (with modified ridit scores), controlling for randomization strata with no adjustment for multiplicity, and *P* values were calculated. Participants with missing AHI score were categorized as missing, and missing data were excluded.

1. *Snoring Analysis*

The sound level (i.e., the decibel increase above background noise) and the percentage of breaths

recorded above a given sound level, were used to select participants who were considered to snore at baseline, in the on-treatment estimand. Treatment groups were compared at week 26 using an analysis of covariance model that included treatment, AHI strata and study as fixed effects, and baseline score as a covariate. The least squares means and treatment difference in least squares means were reported along with the corresponding 95% CI and the *P* values for the treatment group comparison. Missing data were assumed to be MAR and maintained as missing.

1. *Other Exploratory Endpoints Provided in This Report*

Additional protocol-specified exploratory endpoints provided in this report are:

- Change from baseline in non–rapid eye movement (NREM) AHI at week 26
- Change from baseline in REM AHI at week 26, excluding participants whose PSG had <10 minutes of REM sleep
- Change from baseline in supine AHI (AHI_S_) at week 26, excluding participants whose PSG had <10 minutes of supine sleep
- For the 3 endpoints listed above (NREM AHI, REM AHI, and AHI_S_), the analysis was conducted on the on-treatment estimand using a restricted maximum likelihood–based MMRM in combination with the Newton-Raphson algorithm. Analyses included treatment, AHI strata, time, and treatment by time interaction as fixed effects and baseline score as a covariate. An unstructured covariance matrix was used to model the within-participant error. If the model failed to converge with an unstructured covariance matrix, a compound symmetric covariance matrix was used. When structured variance-covariance matrix was used to enable the model to converge, the “sandwich” estimator of the variance-covariance matrix was employed. The Kenward-Roger approximation was used to estimate the denominator degrees of freedom. The analysis was implemented using the MIXED procedure in SAS. The least squares means and treatment difference in least squares means for week 26 were reported along with the corresponding 95% CIs and the *P* values for the treatment group comparisons. Missing data were assumed to be MAR and maintained as missing
- Change from baseline in 2 arousal indices (arousal index - respiratory [ARIR] and arousal index - total [ARI]): descriptive summary of the observed actual and change from baseline values at week 26 by treatment arm

1. *Other* *Exploratory Endpoints Not Provided in This Report*

Additional protocol-specified exploratory endpoints not provided in this report were:

- Percentage of participants with AHI <15, <10, and <5 at weeks 4 and 26
- Change from baseline in AHI based on 3% hypopnea desaturation plus arousals (AHI_3a_) at weeks 4 and 26
- Change from baseline in hypoxic burden (HB) based on 3% hypopnea desaturation (HB_3a_) and oxygen desaturation index (ODI) based on 4% hypopnea desaturation (ODI_4_) at weeks 4 and 26
- Change from baseline in TST and in percentage of TST, in sleep stages:
  - Sleep efficiency (SE)
  - Wake after sleep onset (WASO)
  - Sleep onset latency (SOL)

1. *Exploratory Cohort*

An additional exploratory cohort of up to 100 participants taking a concomitant glucagon-like peptide-1 (GLP-1) agonist specifically for weight loss was to be randomized 1:1 to AD109 or placebo. However, only 7 participants were enrolled (as of March 14, 2025); therefore, no data on this exploratory cohort were presented in the manuscript.

1. **Safety Assessments**

All safety analyses were based on the safety analysis set (all randomized participants who received ≥1 dose of AD109 or placebo). Safety analyses included adverse event frequency, intensity (mild, moderate, severe), and relationship to study drug (related and unrelated).

The assessment of intensity for each adverse event and serious adverse event were reported during the study by the investigator and assigned to one of the following categories: mild, an event that was easily tolerated by the participant, caused minimal discomfort, and did not interfere with everyday activities; moderate, an event that caused sufficient discomfort and interfered with normal everyday activities; and severe, an event that prevented normal everyday activities. The assessment of causality for each adverse event and serious adverse event were reported during the study by the investigator. All adverse events that occurred after the first dose of study drug (i.e., date of onset was on or after the first dose of study drug) and on or before the end of treatment (last dose of study drug) + 7 days were considered treatment-emergent adverse events for the purpose of reporting. Adverse events and serious adverse events were actively collected from randomization until week 28. Additionally, adverse events and serious adverse events were collected after the end of individual patient participation in the study. Investigators were not obligated to actively seek adverse events or serious adverse events after the conclusion of study participation. However, if the investigator learned of any serious adverse event, including a death, at any time after a participant was discharged from the study, and the investigator considered the event to be reasonably related to the study drug or study participation, the investigator notified the sponsor.

- Percentage change from baseline in total sleep time (TST) in sleep stages (E7) in the safety analysis set: Stage N1% (N1P), Stage N2% (N2P), Stage N3% (N3P), Stage R% (RP): descriptive summary of the observed actual and change from baseline values (expressed as percentages of total sleep time) at week 26 by treatment arm

1. **Post Hoc Analyses**

The following analyses were not prespecified in the protocol and were, hence, post hoc:

- The subgroup analyses of AHI, PROMIS-Fatigue, and HB by baseline ESS (<10 vs. ≥10)
- The statistical analysis of the change in OSA severity category from baseline to week 26 using the LOCF approach for the imputation of the missing data

1. **Changes in the Conduct of the Study**

The eligibility criterion of enrolling participants with AHI of ≥5 events/h was subsequently changed to ≥10 to ≤45 events/h to avoid over-representation of participants with very severe or very mild OSA. The eligible AHI range was narrowed to ≥5 to ≤45 events/h on February 7, 2024; 100 participants were randomized using this range. The AHI range was narrowed to ≥10 to ≤45 events/h on March 22, 2024; 518 participants were randomized using this range.

During the course of the study, the order of endpoints was changed to align with recent regulatory approvals for the treatment of OSA related to obesity. Initially, the primary endpoint was the proportion of participants with ≥50% reduction in AHI at week 26 and the change in AHI from baseline to week 26 was an exploratory endpoint. During the course of the study, the change from baseline to week 26 in AHI became the primary endpoint and the proportion of participants with ≥50% reduction in AHI at week 26 became a key secondary endpoint. The change from baseline to week 26 in ODI was switched from an exploratory endpoint to a key secondary endpoint. The order of testing of the secondary endpoints to preserve multiplicity was changed. These changes in the statistical analysis plan were made prior to locking of the study database or any unblinding of treatment assignment. Additional analyses for AHI_3a_ were added in the same way as AHI. The following endpoints were added as exploratory efficacy endpoints in the statistical analysis plan, which are not included in the protocol:

- Percentage of participants with AHI <15, <10, and <5 at weeks 4 and 26
- Proportion of participants with AHI reduction of ≥30% to ≥90% by 10% increments at weeks 4 and 26
- Change from baseline in AHI, AHI_3a_, HB_3a_, and ODI_4_ at weeks 4 and 26
- NREM AHI at weeks 4 and 26
- REM AHI at weeks 4 and 26, excluding participants whose PSG had <10 minutes of REM sleep
- Change from baseline in AHI_S_ at weeks 4 and 26, excluding participants whose PSG had <10 minutes of supine sleep
- Change from baseline in TST and in percentage of TST, in sleep stages (SE; WASO; SOL; N1P, N2P, N3P, RP)
- Change from baseline in 2 arousal indices (ARIR and ARI)
- Proportion of participants with ≥50% reduction in AHI at weeks 4 and 26
- Change from baseline to weeks 4 and 26 in OSA severity category (none, mild, moderate, and severe)
- Proportion of participants with a reduction in breaths with snoring

The following endpoints were removed due to redundancy:

- Proportion of participants with ≥50% reduction in AHI_3a_ at weeks 4 and 26
- Proportion of participants with ≥50% reduction in ODI at weeks 4 and 26

**REFERENCES**

**E1.** Berry RB, Abreu AR, Krishnan V, Quan SF, Strollo PJ, Malhotra RK. A transition to the American Academy of Sleep Medicine-recommended hypopnea definition in adults: initiatives of the Hypopnea Scoring Rule Task Force. *J Clin Sleep Med* 2022;18:1419-1425.

**E2.** Schweitzer PK, Taranto-Montemurro L, Ojile JM, Thein SG, Drake CL, Rosenberg R, *et al*. The combination of aroxybutynin and atomoxetine in the treatment of obstructive sleep apnea (MARIPOSA): a randomized controlled trial. *Am J Respir Crit Care Med* 2023;208:1316–1327.

**E3.** Azarbarzin A, Sands SA, Stone KL, Taranto-Montemurro L, Messineo L, Terrill PI, *et al*. The hypoxic burden of sleep apnoea predicts cardiovascular disease-related mortality: the Osteoporotic Fractures in Men Study and the Sleep Heart Health Study. *Eur Heart J* 2019;40:1149–1157.

**E4.** O'Kelly M, Ratitch B. Clinical trial with missing data: a guide for practitioners. West Sussex, United Kingdom: John Wiley & Sons, Ltd; 2014.

**E5.** Mantel N, Haenszel W. Statistical aspects of the analysis of data from retrospective studies of disease. *J Natl Cancer Inst* 1959;22:719–748.

**E6.** Sato T, Greenland S, Robins JM. On the variance estimator for the Mantel-Haenszel risk difference. *Biometrics* 1989;45:1323–1324.

**E7.** Malhotra RK. AASM scoring manual 3: a step forward for advancing sleep care for patients with obstructive sleep apnea. *J Clin Sleep Med* 2024;20:835–836.

**E8.** Breslow NE, Day NE. Statistical methods in cancer research. Volume I - The analysis of case-control studies. *IARC Sci Publ* 1980;32:5–338.

**Supplementary Figures**

**Figure Legends**

**Figure E1.** SynAIRgy study design. The study enrolled participants with obstructive sleep apnea. AHI = apnea-hypopnea index based on 4% hypopnea desaturation; AROX = aroxybutynin; ATO = atomoxetine; HB = hypoxic burden based on 4% hypopnea desaturation; ODI = oxygen desaturation index based on 3% hypopnea desaturation; PROMIS = Patient-Reported Outcomes Measurement Information System; PSG = polysomnography; and R = randomized. *Participants with AHI ≥5 events/h were initially eligible; subsequently, the eligible AHI range was narrowed to 10–45 events/h to avoid over-representation of participants with very mild or very severe obstructive sleep apnea. ^†^Standard overnight PSG was performed according to the American Academy of Sleep Medicine (AASM) scoring manual version 3 (E7).

**Figure E2.** Baseline distribuiton of AHI (ITT).

AHI = apnea-hypopnea index based on 4% desaturation; ITT = intent to treat.

**Figure E3.** Subgroup analyses of the mean change in AHI from baseline to week 26 (primary endpoint) by intent to treat (*A*) and on-treatment estimands (*B*). AHI = apnea-hypopnea index based on 4% hypopnea desaturation; BMI = body mass index; ESS = Epworth Sleepiness Scale; ITT = intent to treat; LS = least squares; and PROMIS = Patient-Reported Outcomes Measurement Information System. *Post hoc analysis; data missing from 5 participants (AD109, *n* = 3; placebo, *n* = 2). ^†^Other race category includes American Indian or Alaska Native, Native Hawaiian or other Pacific Islander, and other.

**Figure E4.** Mean percent reduction in HB from baseline to weeks 4 and 26 (secondary key efficacy endpoint) by intent to treat (*A*) and on-treatment estimands (*B*). Due to skewed distribution, HB was analyzed and modeled on the natural log scale; the least squares mean estimated relative change from baseline from mixed model for repeated measurements analysis were back transformed to original scale of relative change from baseline and expressed as a percentage for presentation. Log (0.01) was used if HB was reported as 0. Error bars represent 95% CIs. *****P*<0.0001 vs. placebo. HB = hypoxic burden based on 4% hypopnea desaturation; ITT = intent to treat.

**Figure E5.** Subgroup analyses of the fold change in HB from baseline to week 26 (secondary key efficacy endpoint) by intent to treat (*A*) and on-treatment estimands (*B*). AHI = apnea-hypopnea index based on 4% hypopnea desaturation; BMI = body mass index; ESS = Epworth Sleepiness Scale; ITT = intent to treat; LS = least squares; and PROMIS = Patient-Reported Outcomes Measurement Information System. *Post hoc analysis; data missing from 5 participants (AD109, *n* = 3; placebo, *n* = 2). ^†^Other race category includes American Indian or Alaska Native, Native Hawaiian or other Pacific Islander, and other.

**Figure E6.** Select subgroup analyses of the mean change in PROMIS-Fatigue T-score from baseline to week 26 (secondary key efficacy endpoint) by intent to treat (*A*) and on-treatment estimands (*B*). ESS = Epworth Sleepiness Scale; ITT = intent to treat; LS = least squares; and PROMIS = Patient-Reported Outcomes Measurement Information System. *Post hoc analysis; data missing from 5 participants (AD109, *n* = 3; placebo, *n* = 2).

**Figure E7.** Proportion of participants with change in OSA severity category from baseline to week 26 (exploratory endpoint; on-treatment estimand). Data collected after treatment discontinuation was set to missing. Missing data were imputed using the last observation carried forward approach (post hoc analysis). AHI = apnea-hypopnea index based on 4% hypopnea desaturation; OSA = obstructive sleep apnea.

**Figure E8**. Panel represents model-estimated change from baseline in AHI at week 26 as a function of baseline AHI, derived from the prespecified primary endpoint ANCOVA model including baseline AHI as a covariate. Estimates are shown for AD109 and placebo arms in the ITT, and AD109 in the on-treatment estimands. Predicted absolute changes were obtained by back-transforming model-based relative clinical response to the original AHI. AHI = apnea-hypopnea index based on 4% hypopnea desaturation; ANCOVA = analysis of covariance; ITT = intent to treat.

**Figure E9**. Mean change from baseline to weeks 4 and 26 in AHI (*A*), ODI (*B*), and HB (C) in the on-treatment estimand. This post hoc analysis, aimed to assess tachyphylaxis, was conducted in a subset of participants who had data at both time points. In the AD109 arm, 61 participants were excluded from this analysis and in the placebo arm, 37 participants were excluded because they did not have data at both time points. Error bars represent 95% CIs. AHI = apnea-hypopnea index based on 4% hypopnea desaturation; HB = hypoxic burden based on 4% hypopnea desaturation; ODI = oxygen desaturation index based on 3% desaturation.

**Figure 10.** Timing of study discontinuations in the AD109 arm (safety analysis set). AE = adverse event.

**Figures**

**
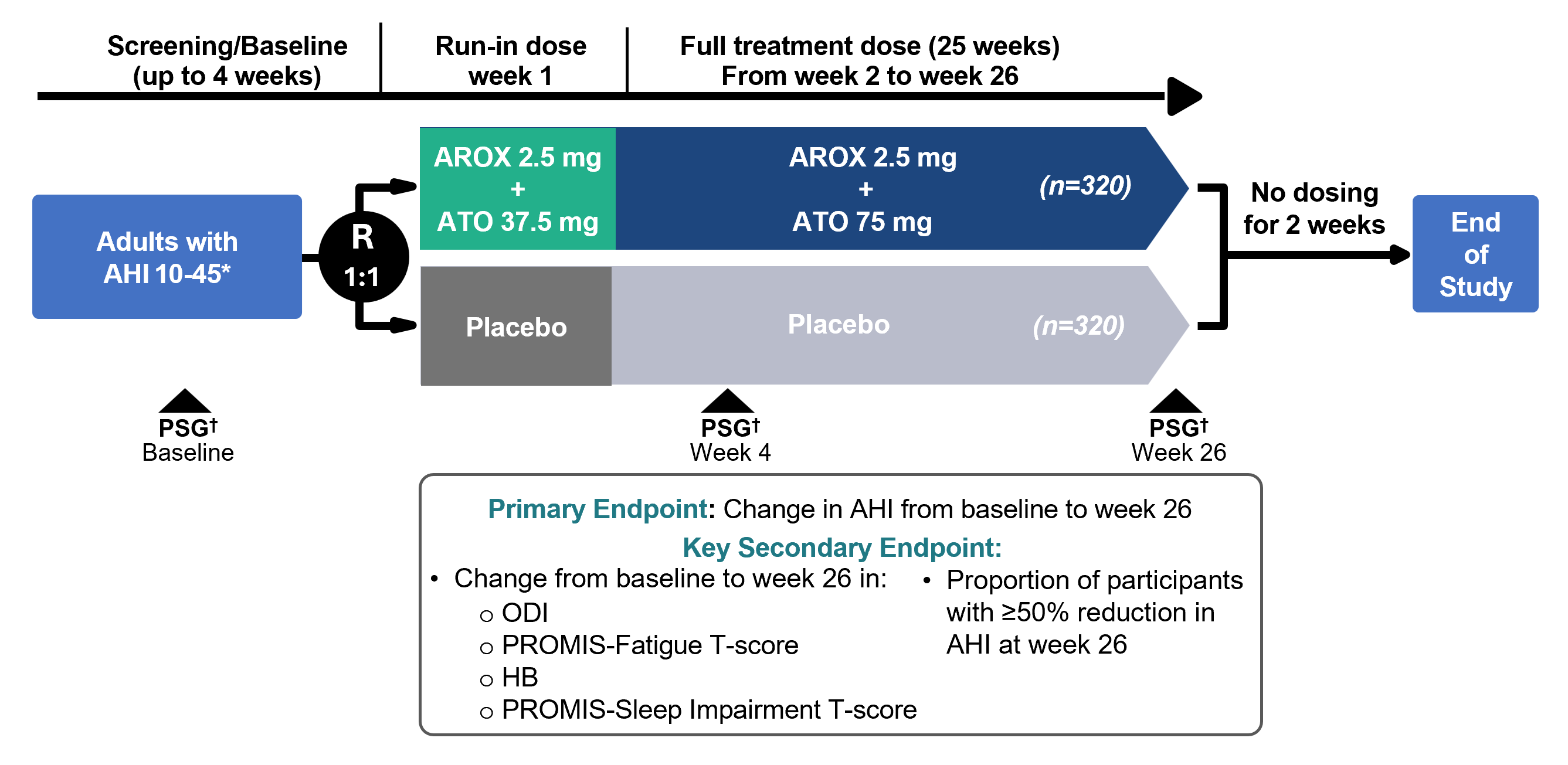
**

**Figure E1.** SynAIRgy study design.

**
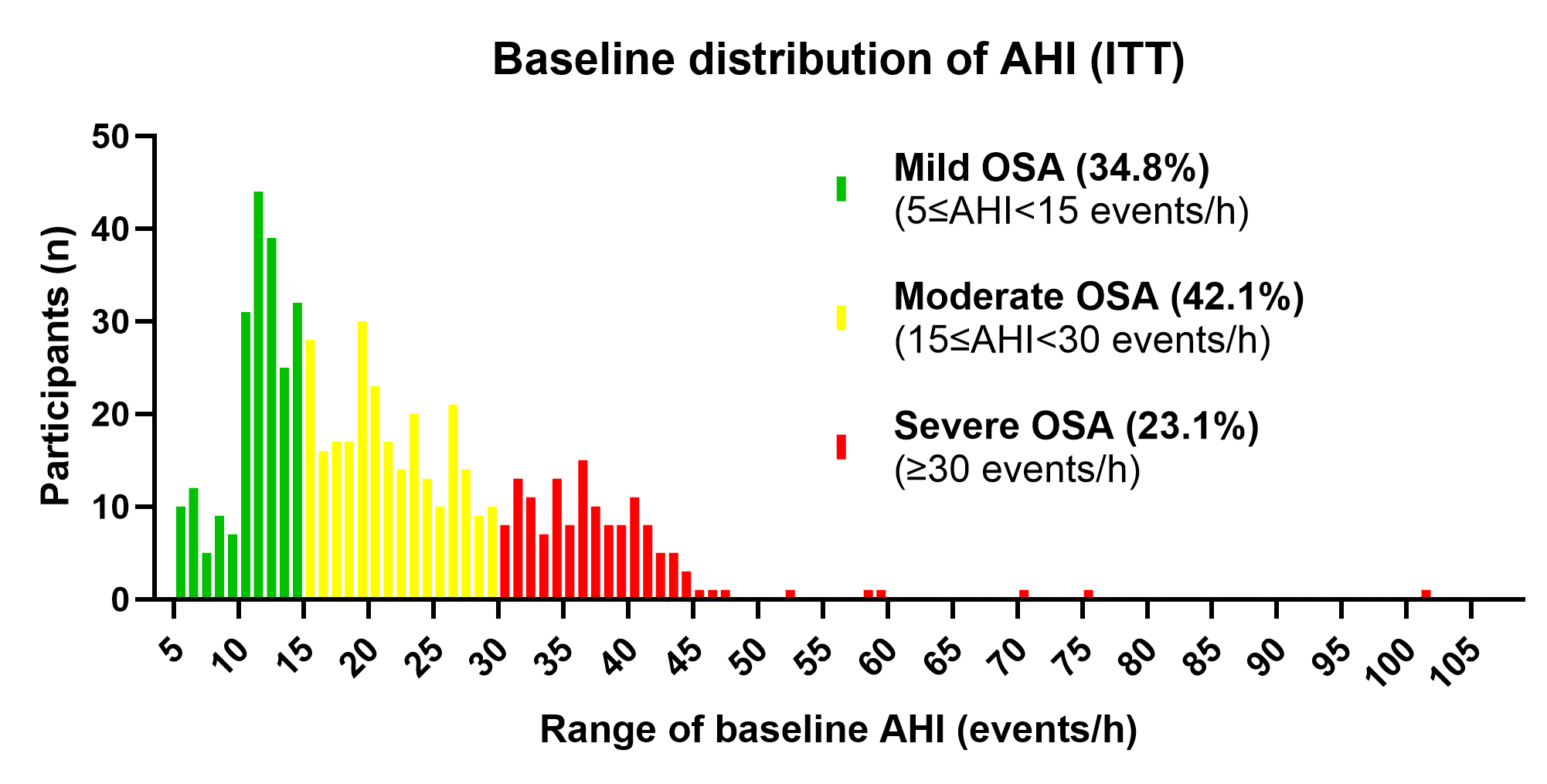
**

**Figure E2.** Baseline distribuiton of AHI (ITT).

**
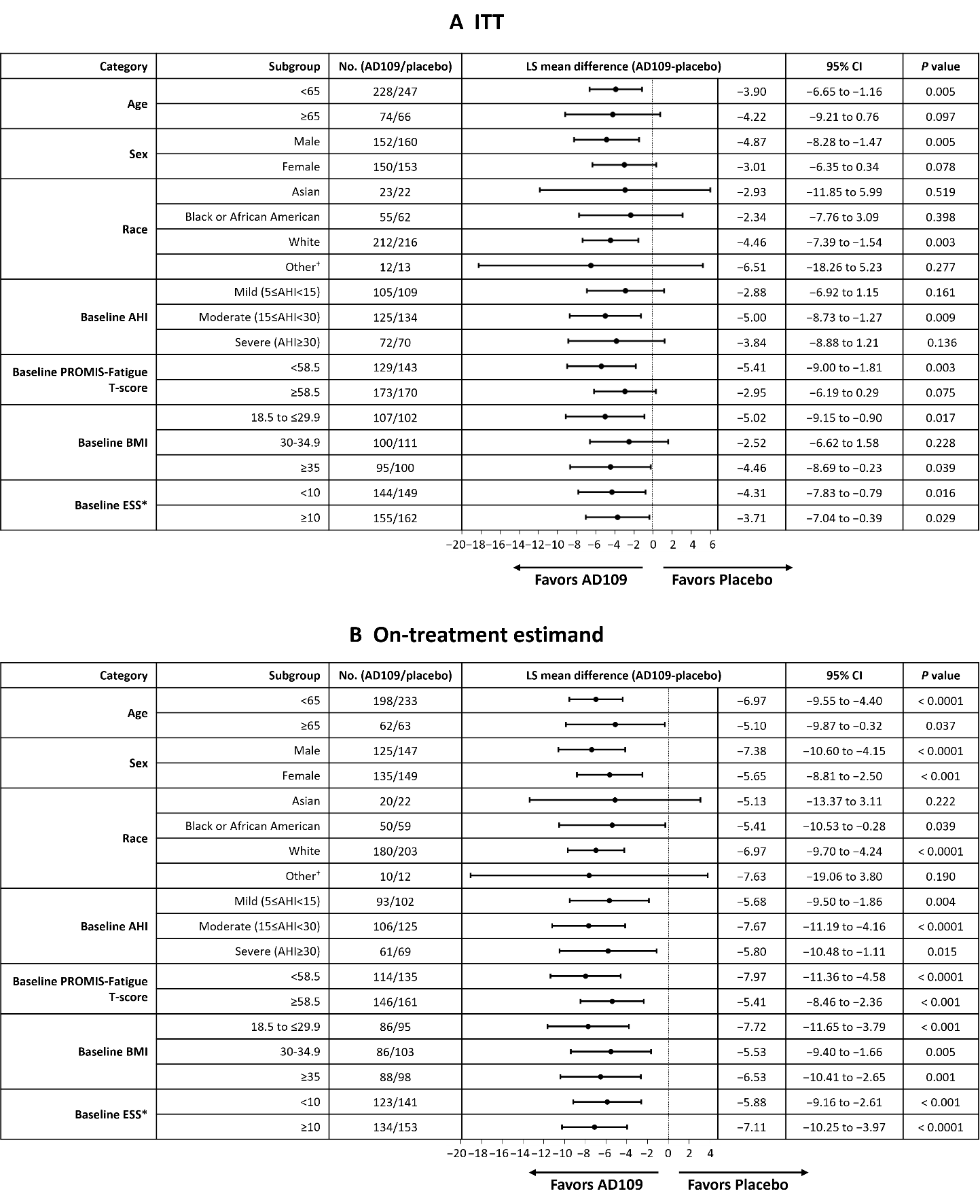
**

**Figure E3.** Subgroup analyses of the mean change in AHI from baseline to week 26 (primary endpoint) by intent to treat (*A*) and on-treatment estimands (*B*).


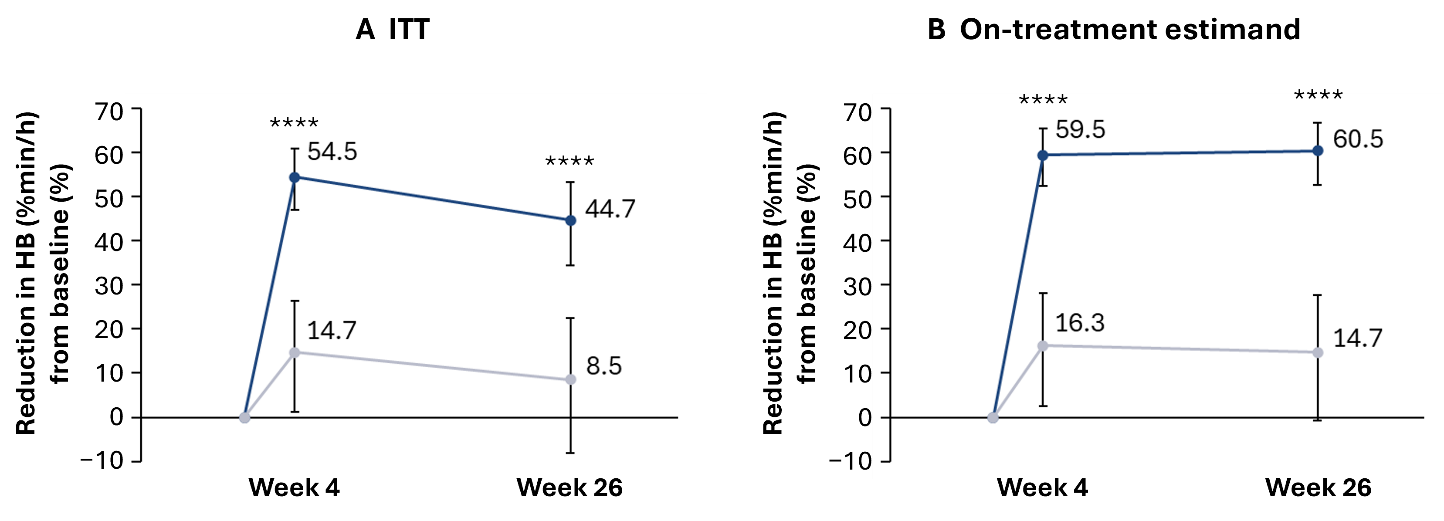


**Figure E4.** Mean percent reduction in HB from baseline to weeks 4 and 26 (secondary key efficacy endpoint) by intent to treat (*A*) and on-treatment estimands (*B*).

**
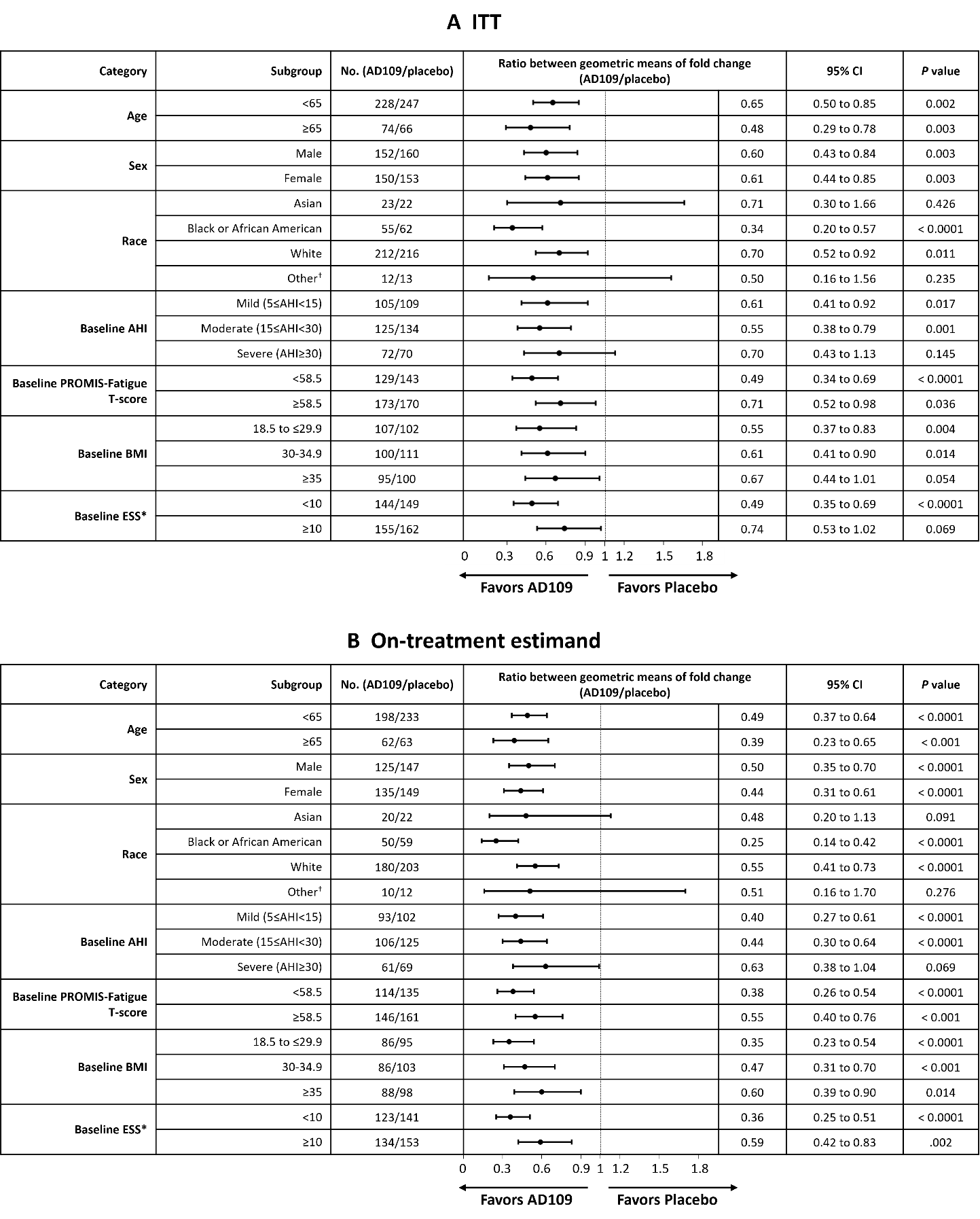
**

**Figure E5.** Subgroup analyses of the fold change in HB from baseline to week 26 (secondary key efficacy endpoint) by intent to treat (*A*) and on-treatment estimands (*B*).

**
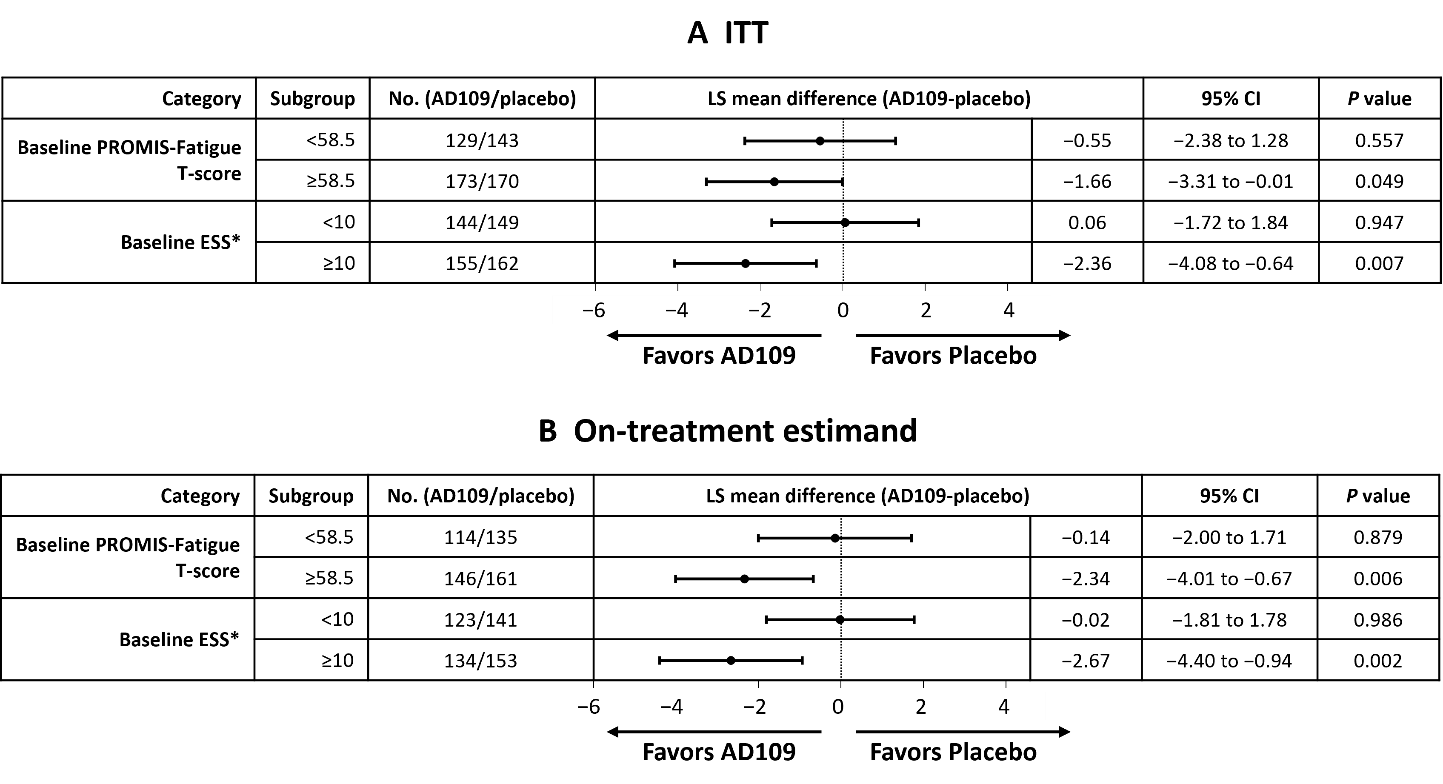
**

**Figure E6.** Select subgroup analyses of the mean change in PROMIS-Fatigue T-score from baseline to week 26 (secondary key efficacy endpoint) by intent to treat (*A*) and on-treatment estimands (*B*).


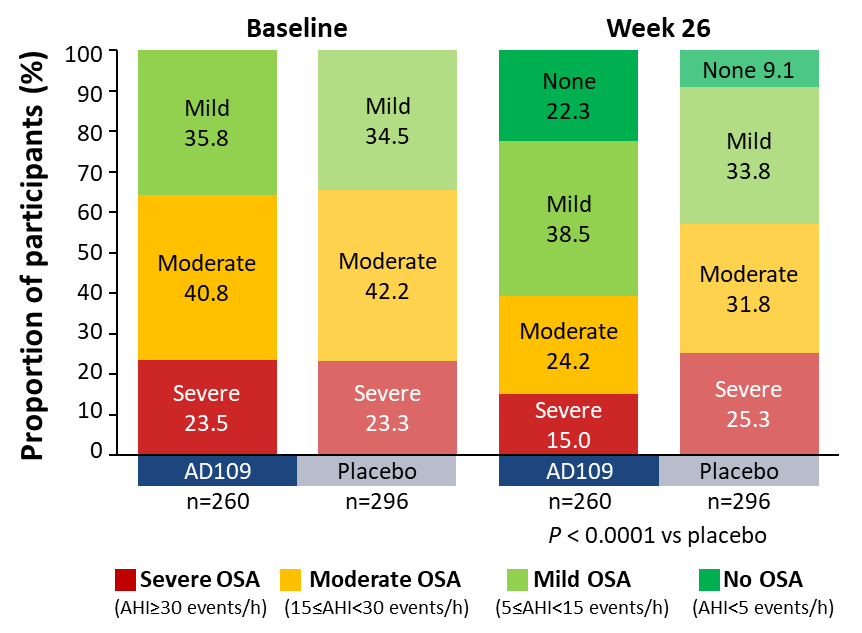


**Figure E7.** Proportion of participants with change in OSA severity category from baseline to week 26 (exploratory endpoint; on-treatment estimand).


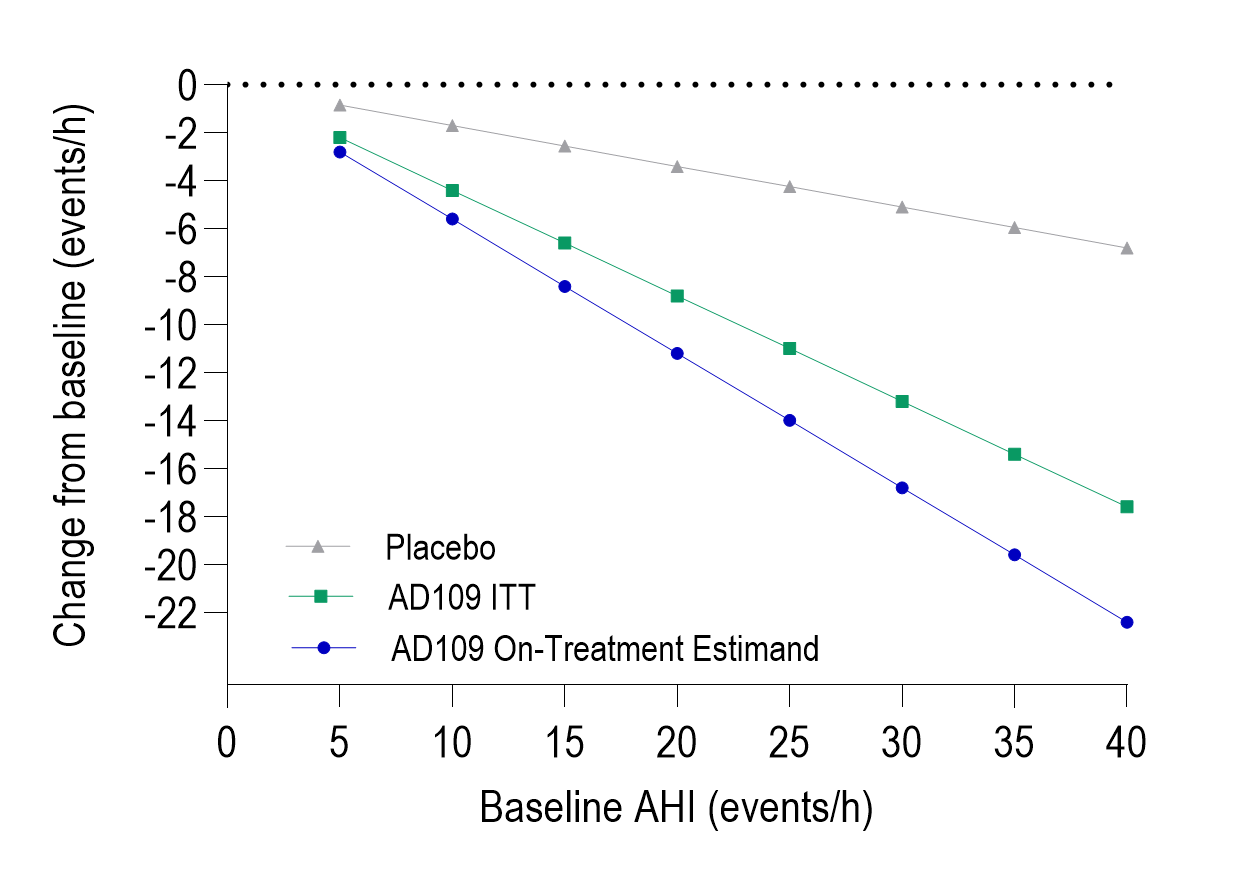


**Figure E8**. Panel represents model-estimated change from baseline in AHI at week 26 as a function of baseline AHI, derived from the prespecified primary endpoint ANCOVA model including baseline AHI as a covariate.

**
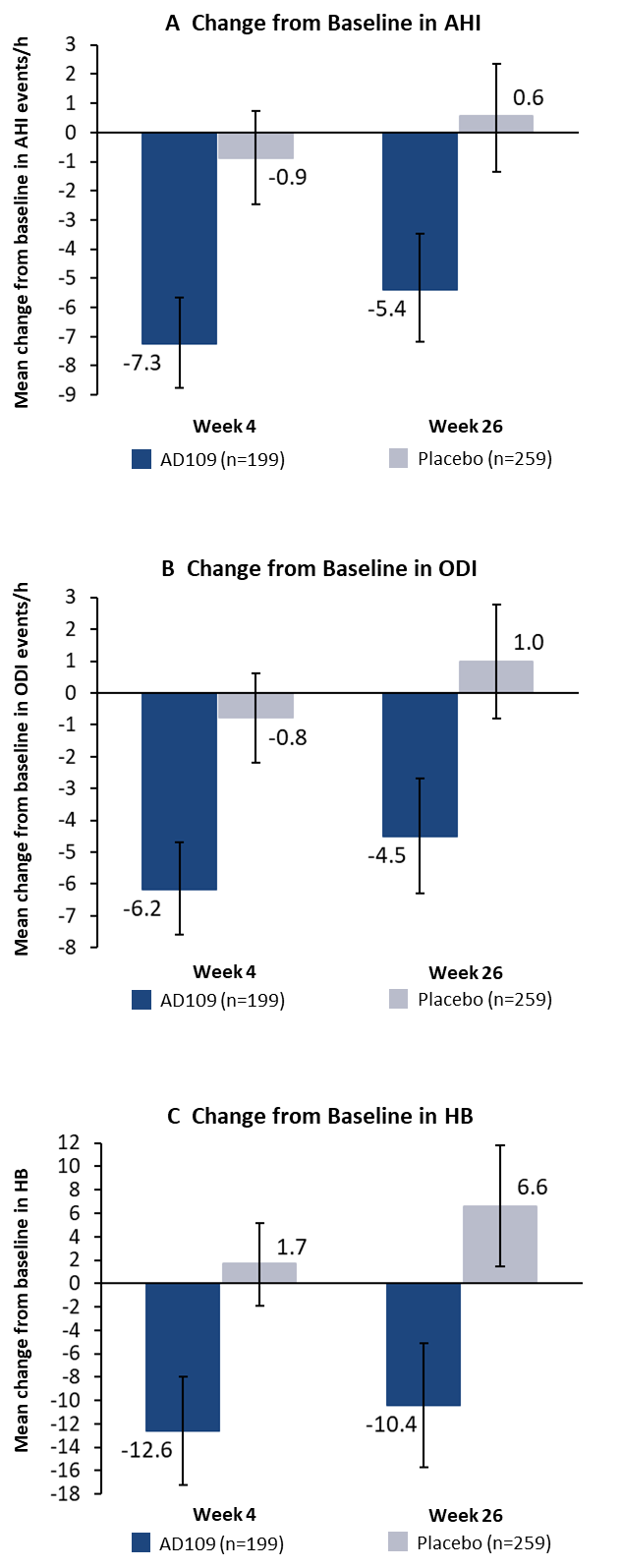
**

**Figure E9**. Mean change from baseline to weeks 4 and 26 in AHI (*A*), ODI (*B*), and HB (C) in the on-treatment estimand.

**
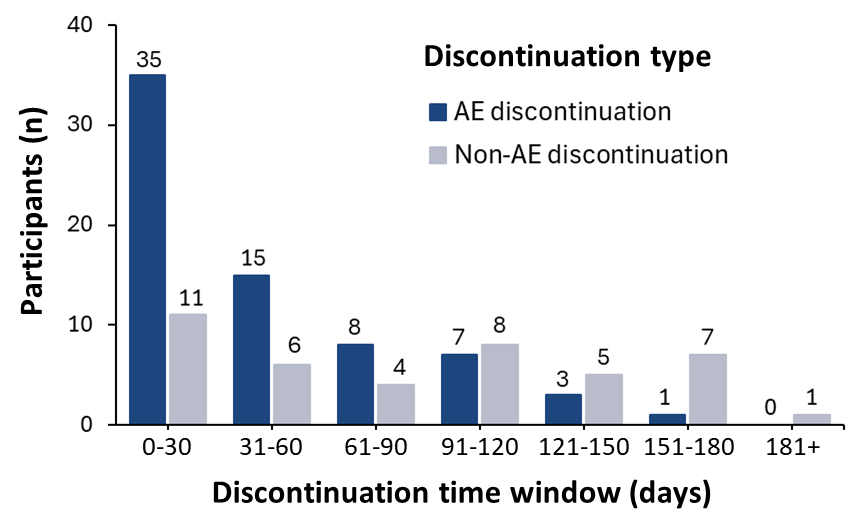
**

**Figure 10.** Timing of study discontinuations in the AD109 arm (safety analysis set).

**Supplementary Tables**

**Table E1. Observed Values for Primary and Key Secondary Endpoints at Baseline and at Week 26 (Intent to Treat and On-Treatment Estimand)**

| **ITT (*N* = 615)** | | | | | | | |
| --- | --- | --- | --- | --- | --- | --- | --- |
|  | **AD109 (*n* = 302)** | | | **Placebo (*n* = 313)** | | | |
|  | **Baseline** | **Week 26** | | **Baseline** | | **Week 26** | |
| **Primary Efficacy Endpoint** | | | | | | | |
| **AHI, Median (IQR) events/h [*n*]** | 19.8  (12.8 to 28.9) | 13.3  (6.5 to 24.2)  [258] | | 19.1  (12.7 to 28.3) | | 17.7  (9.0 to 30.5)  [272] | |
| **Key Secondary Efficacy Endpoints** | | | | | | | |
| **ODI, Median (IQR), events/h [*n*]** | 27.3  (19.4 to 37.6) | | 20.5  (10.9 to 32.3)  [258] | | 26.9  (19.1 to 36.0) | | 24.0  (14.2 to 38.3)  [272] |
| **PROMIS-Fatigue T-score, Mean (SD) [*n*]** | 59.2 (7.0) | | 51.4 (8.3)  [255] | | 58.9 (6.7) | | 52.8 (8.1)  [275] |
| **HB, Median (IQR), %min/h [*n*]** | 33.7  (19.1 to 56.5) | | 23.2  (7.3 to 48.2)  [258] | | 28.8  (16.8 to 53.2) | | 28.4  (12.4 to 60.3)  [272] |
| **PROMIS-Sleep Impairment T-score, Mean (SD) [*n*]** | 58.9 (7.2)  [299] | | 50.8 (8.6)  [256] | | 58.6 (7.4)  [309] | | 51.8 (9.1)  [275] |
| **On-Treatment Estimand (*N* = 556)** | | | | | | | |
|  | **AD109 (*n* = 260)** | | | | **Placebo (*n* = 296)** | | |
|  | **Baseline** | | **Week 26** | | **Baseline** | | **Week 26** |
| **Primary Efficacy Endpoint** | | | | | | | |
| **AHI, Median (IQR) events/h** | 19.3  (12.7 to 28.7) | | 10.6  (5.3 to 20.0) | | 19.2  (12.9 to 29.1) | | 18.0  (9.2 to 30.5) |
| **Key Secondary Efficacy Endpoints** | | | | | | | |
| **ODI, Median (IQR), events/h** | 26.0  (19.0 to 37.2) | | 17.5  (9.5 to 29.5) | | 27.2  (18.9 to 36.9) | | 24.8  (14.4 to 38.6) |
| **PROMIS-Fatigue T-score, Mean (SD)** | 59.2 (7.2) | | 51.6 (8.4) | | 58.9 (6.5) | | 52.7 (8.1) |
| **HB, Median (IQR), %min/h** | 33.0  (19.0 to 54.2) | | 18.3  (5.4 to 38.7) | | 29.2  (16.9 to 54.1) | | 29.1  (13.0 to 60.8) |
| **PROMIS-Sleep Impairment T-score, Mean (SD)** | 58.7 (7.3)  [257] | | 50.9 (8.9)  [259] | | 58.5 (7.2)  [292] | | 51.7 (8.9) |

*Definition of abbreviations:* AHI = apnea-hypopnea index based on 4% hypopnea desaturation; HB = hypoxic burden based on 4% desaturation; IQR = interquartile range; ITT = intent to treat; ODI = oxygen desaturation index based on 3% desaturation; PROMIS = Patient-Reported Outcomes Measurement Information System; SD = standard deviation

**Table E2.** Snoring Analysis (Exploratory Endpoint; On-Treatment Estimand)*

| **On-Treatment Estimand (*N* = 556)** | | | | | | |
| --- | --- | --- | --- | --- | --- | --- |
| **Proportion of breaths with snoring,^†^ median (IQR) [*n*]** | | | | **Proportion of breaths with snoring, LS mean (95% CI)** | | **Estimated treatment difference of LS means [AD109-placebo]**  **(95% CI) and *P* value** |
| **AD109 (*n* = 125)** | | **Placebo (*n* = 149)** | |  |  |  |
| **Baseline** | **Week 26** | **Baseline** | **Week 26** | **AD109** | **Placebo** |  |
| 36.0  (24.0 to 57.5)  [125] | 10.6  (2.8 to 30.2)  [113] | 39.7  (24.4 to 57.1)  [149] | 31.4  (8.1 to 51.8)  [131] | 21.6  (17.8 to 25.5) | 31.2  (27.7 to 34.8) | −9.6  (−14.8 to −4.4)  *P* = 0.0004 |

*Definition of abbreviations:* CI = confidence interval; LS = least squares.

*Analysis was prespecified for on-treatment estimand.

^†^Snoring defined as 25 decibels above background breathing noise for ≥15% of breaths. Missing data were assumed to be missing at random and maintained as missing.

**Table E3.** Treatment effect on AHI and HB at Week 26 Performed with Adjusted Model Including the Change from Baseline in Proportion of REM Sleep (On-Treatment Estimand)

| **On-Treatment Estimand (*N* = 556)** | | | | |
| --- | --- | --- | --- | --- |
|  | **AD109 (n = 260)** | **Placebo (n = 296)** | **Estimated treatment difference**  **(95% CI), (n = 260)** | ***P* value** |
| **Change from baseline in AHI at week 26, LS mean (95% CI), events/h** | | | | |
| Primary model | −6.1 (−7.8 to −4.4) | 0.4 (−1.2 to 2.0) | −6.5 (−8.8 to −4.3) | *P*<0.0001 |
| Model adjusted for change in proportion of REM sleep | −5.6 (−7.7 to −3.6) | 0.7 (−1.1 to 2.4) | −6.3 (−9.0 to −3.6) | *P*<0.0001 |
| Model adjusted for change in BMI | −5.2 (−7.1 to −3.3) | 0.3 (−1.4 to 2.0) | −5.5 (−8.0 to −3.0) | *P*<0.0001 |
| **Percent reduction*** **from baseline in HB at week 26, LS mean (95% CI)** | | | | |
| Primary model | 60.5 (52.8 to 66.9) | 14.7 (−0.7 to 27.8) | 53.6 (41.0 to 63.6) | *P*<0.0001 |
| Model adjusted for change in proportion of REM sleep | 55.6 (45.3 to 64.0) | 22.4 (6.6 to 35.5) | 42.8 (24.0 to 57.0) | *P*<0.001 |

*Definition of abbreviations:* AHI = apnea-hypopnea index based on 4% hypopnea desaturation; CI = confidence interval; HB = hypoxic burden based on 4% hypopnea desaturation; LS = least squares.

*HB was analyzed on the natural log scale and then back transformed to original scale of relative change.

**Table E4.** Summary of Adverse Events Occurring in ≥5%* of Participants in Either Arm, by Severity (Safety Analysis Set)

| **Adverse Event** | **AD109 (*n* = 325)** | | | **Placebo (*n* = 319)** | | |
| --- | --- | --- | --- | --- | --- | --- |
|  | **Mild^‡^** | **Moderate^‡^** | **Severe^‡^** | **Mild^‡^** | **Moderate^‡^** | **Severe^‡^** |
| Any TEAE,^†^ *n* (%) | 150 (46.2) | 65 (20.0) | 15 (4.6) | 94 (29.5) | 45 (14.1) | 10 (3.1) |
| Dry mouth | 98 (30.2) | 14 (4.3) | 3 (0.9) | 27 (8.5) | 2 (0.6) | 0 |
| Insomnia^§^ | 52 (16.0) | 12 (3.7) | 3 (0.9) | 11 (3.4) | 4 (1.3) | 0 |
| Nausea | 30 (9.2) | 6 (1.8) | 1 (0.3) | 2 (0.6) | 0 | 0 |
| Urinary hesitation | 24 (7.4) | 4 (1.2) | 1 (0.3) | 1 (0.3) | 0 | 0 |
| Constipation | 12 (3.7) | 5 (1.5) | 0 | 4 (1.3) | 0 | 0 |
| Somnolence | 12 (3.7) | 5 (1.5) | 0 | 6 (1.9) | 3 (0.9) | 1 (0.3) |

*Definition of abbreviations:* TEAE = treatment-emergent adverse event.

*The ≥5% threshold is based on all-grade TEAEs.

^†^Adverse events were coded by Medical Dictionary for Regulatory Activities v27.0 or higher. A TEAE is defined as an adverse event with an onset or diagnosis after the first dose of study medication and on or before the end of treatment (last dose of study medication +7 days), regardless of causality.

^‡^Based on investigator assessment. Participants reporting more than 1 event were counted only once at the worst intensity. An adverse event with missing intensity was considered severe.

^§^Includes initial insomnia, insomnia, and middle insomnia.

**Table E5.** Summary of Adverse Events Occurring in ≥5%* of Participants in Either Arm, by Relationship (Safety Analysis Set)

| **Adverse Event** | **AD109 (*n* = 325)** | | **Placebo (*n* = 319)** | |
| --- | --- | --- | --- | --- |
|  | **Related^‡^** | **Not related^‡^** | **Related^‡^** | **Not related^‡^** |
| Any TEAE,^†^ *n* (%) | 190 (58.5) | 122 (37.5) | 75 (23.5) | 105 (32.9) |
| Dry mouth | 113 (34.8) | 2 (0.6) | 29 (9.1) | 0 |
| Insomnia^§^ | 64 (19.7) | 3 (0.9) | 12 (3.8) | 3 (0.9) |
| Nausea | 36 (11.1) | 1 (0.3) | 1 (0.3) | 1 (0.3) |
| Urinary hesitation | 28 (8.6) | 1 (0.3) | 1 (0.3) | 0 |
| Constipation | 15 (4.6) | 2 (0.6) | 4 (1.3) | 0 |
| Somnolence | 14 (4.3) | 3 (0.9) | 9 (2.8) | 1 (0.3) |

*Definition of abbreviations:* TEAE = treatment-emergent adverse event.

*The ≥5% threshold is based on all-grade TEAEs.

^†^Adverse events were coded by Medical Dictionary for Regulatory Activities v27.0 or higher. A TEAE is defined as an adverse event with an onset or diagnosis after the first dose of study medication and on or before the end of treatment (last dose of study medication +7 days), regardless of causality.

^‡^Based on investigator assessment. If a participant had more than 1 event, which were different in relationship, the participant was counted once and the related event was considered.

^§^Includes initial insomnia, insomnia, and middle insomnia.
