# Supplementary figures and images for: Aroxybutynin and atomoxetine (AD109) for obstructive sleep apnea: a randomized phase 3 trial (SynAIRgy)
Source: Am J Respir Crit Care Med. 2026 May 18;212(7):1569–84. doi: 10.1093/ajrccm/aamag215 (PMC13318230; doi:10.1093/ajrccm/aamag215)

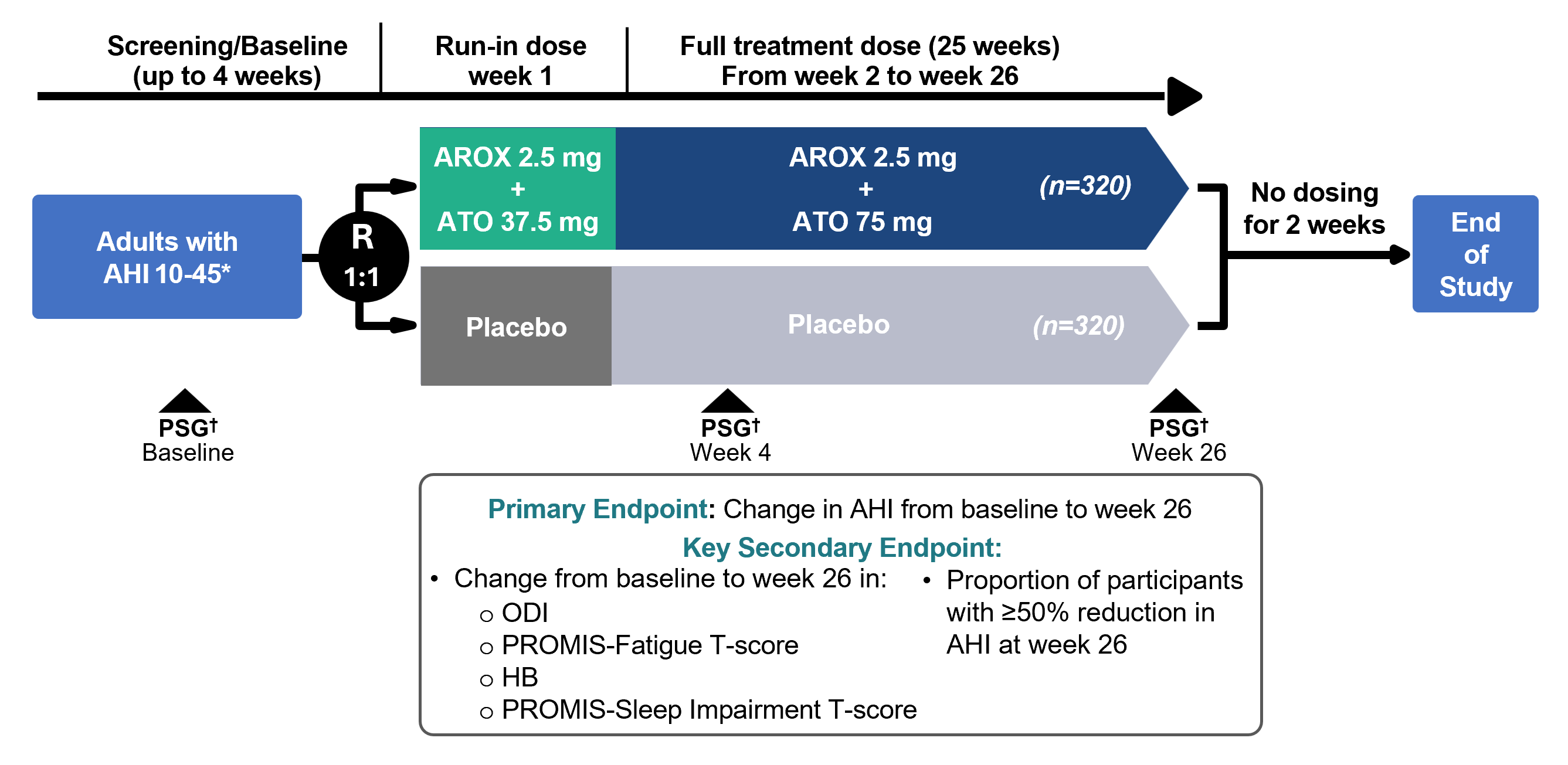

Supplement: aamag215_Supplementary_Data [file aamag215_supplementary_data.zip › Figure E1.png]

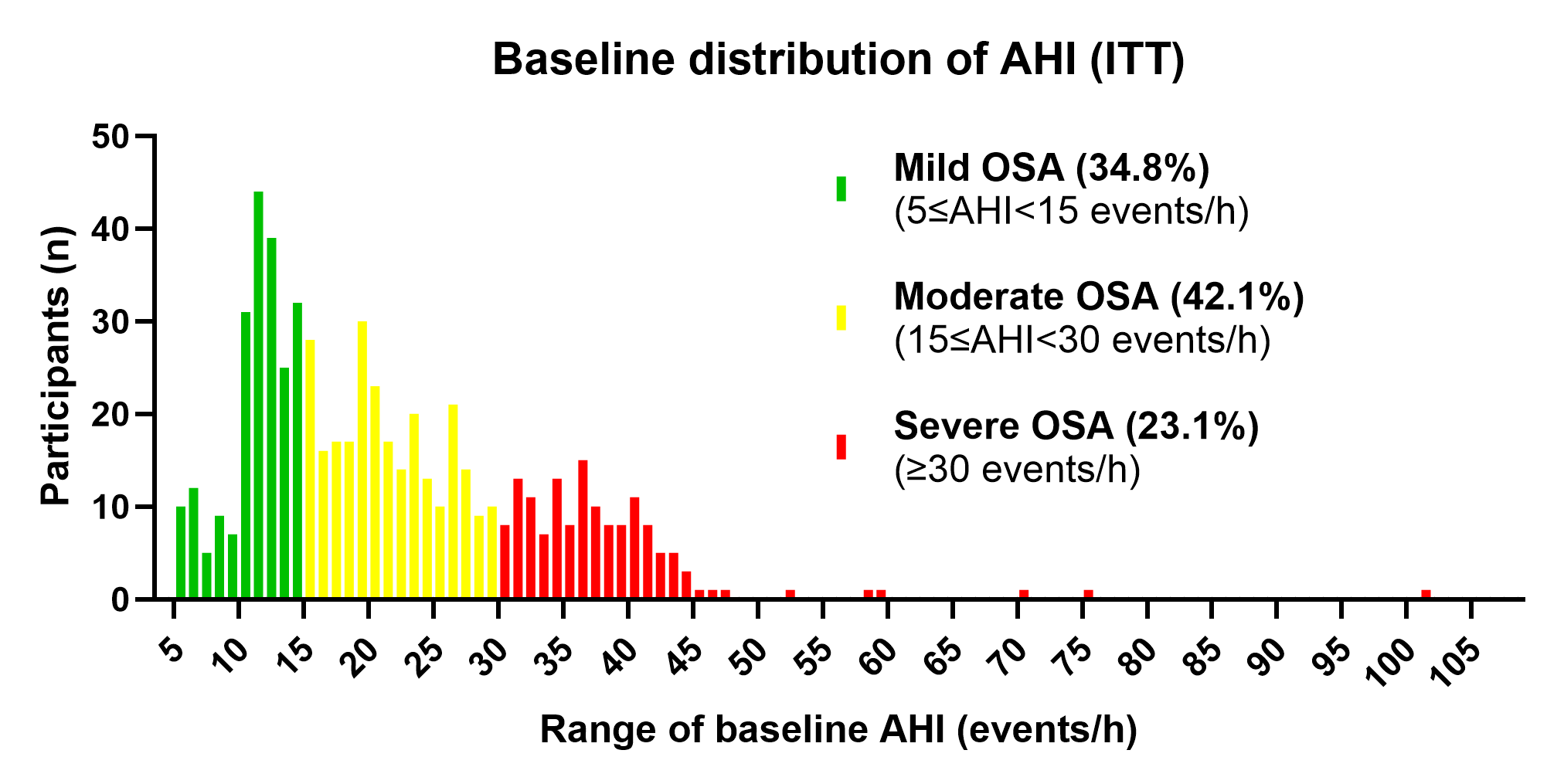

Supplement: aamag215_Supplementary_Data [file aamag215_supplementary_data.zip › Figure E2.png]

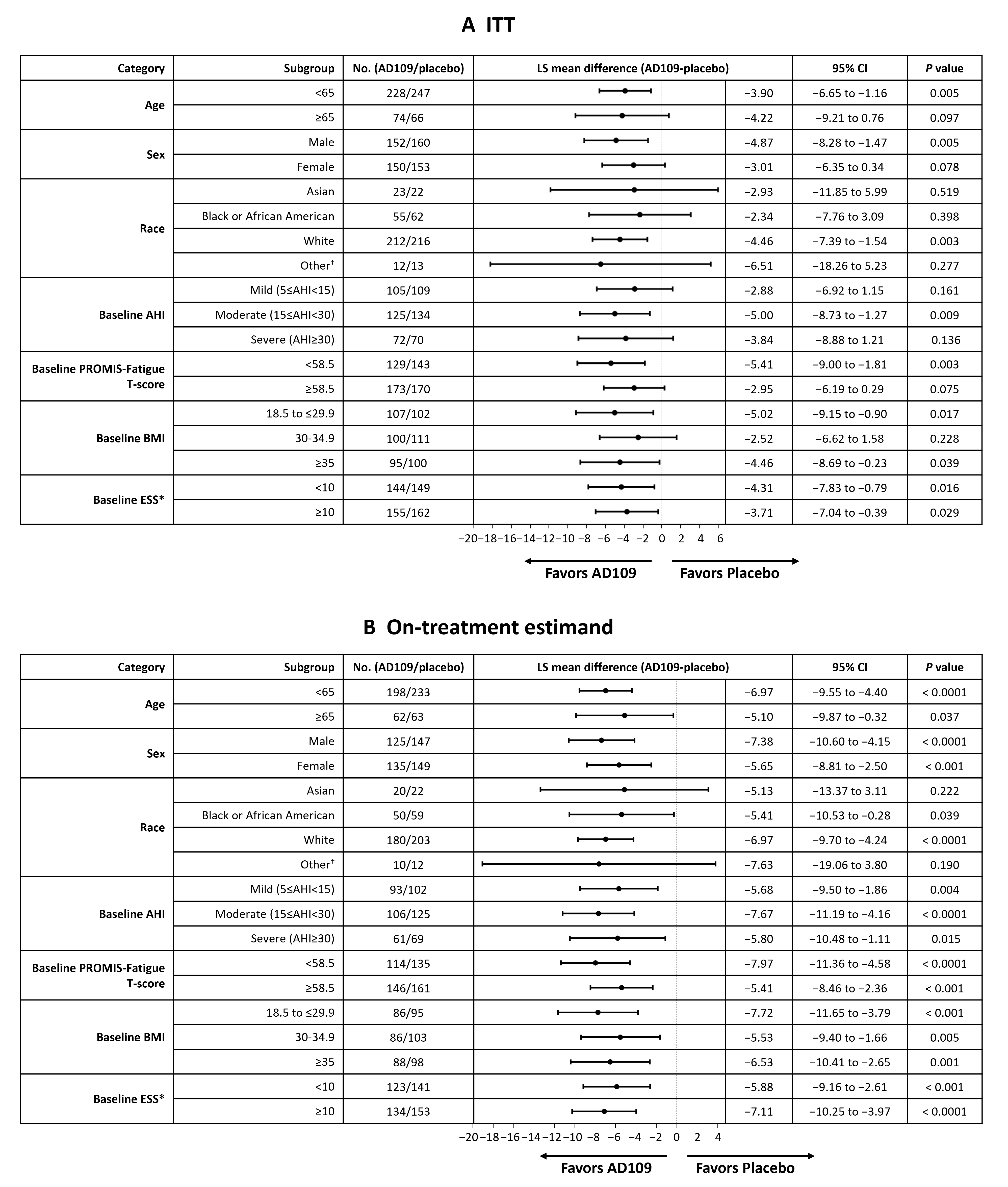

Supplement: aamag215_Supplementary_Data [file aamag215_supplementary_data.zip › Figure E3.png]

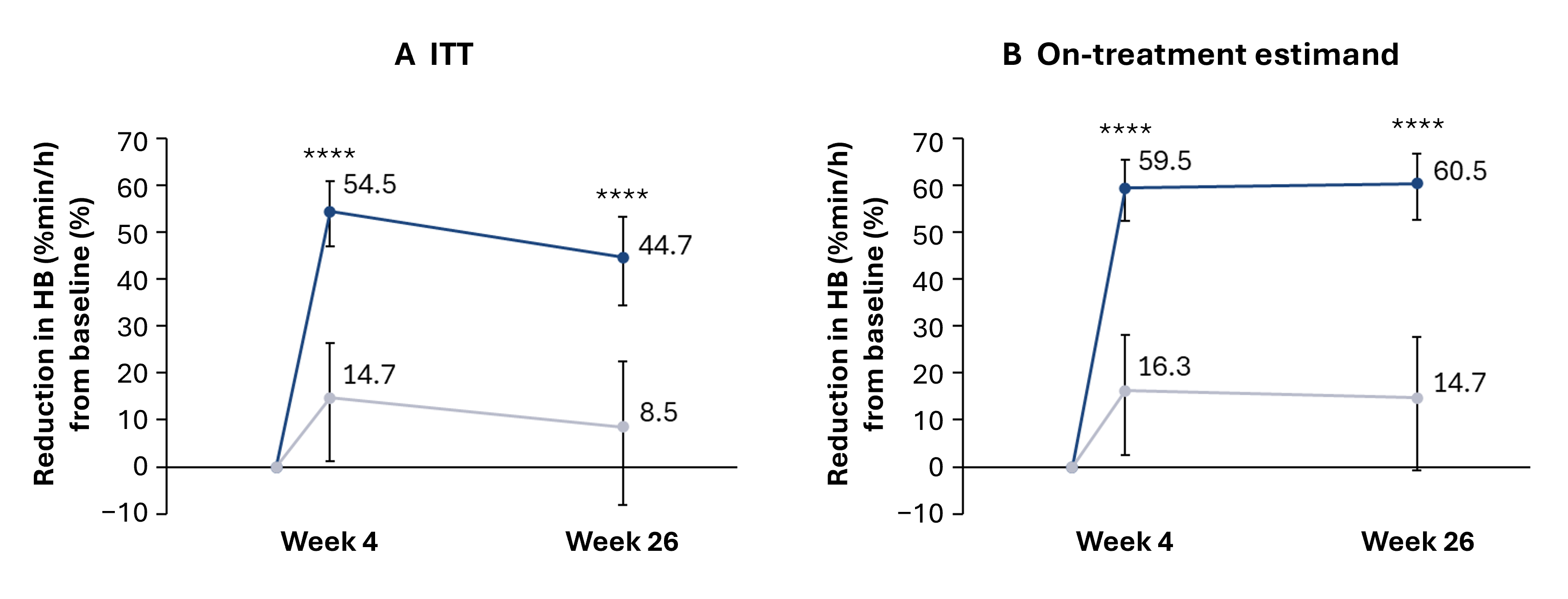

Supplement: aamag215_Supplementary_Data [file aamag215_supplementary_data.zip › Figure E4.png]

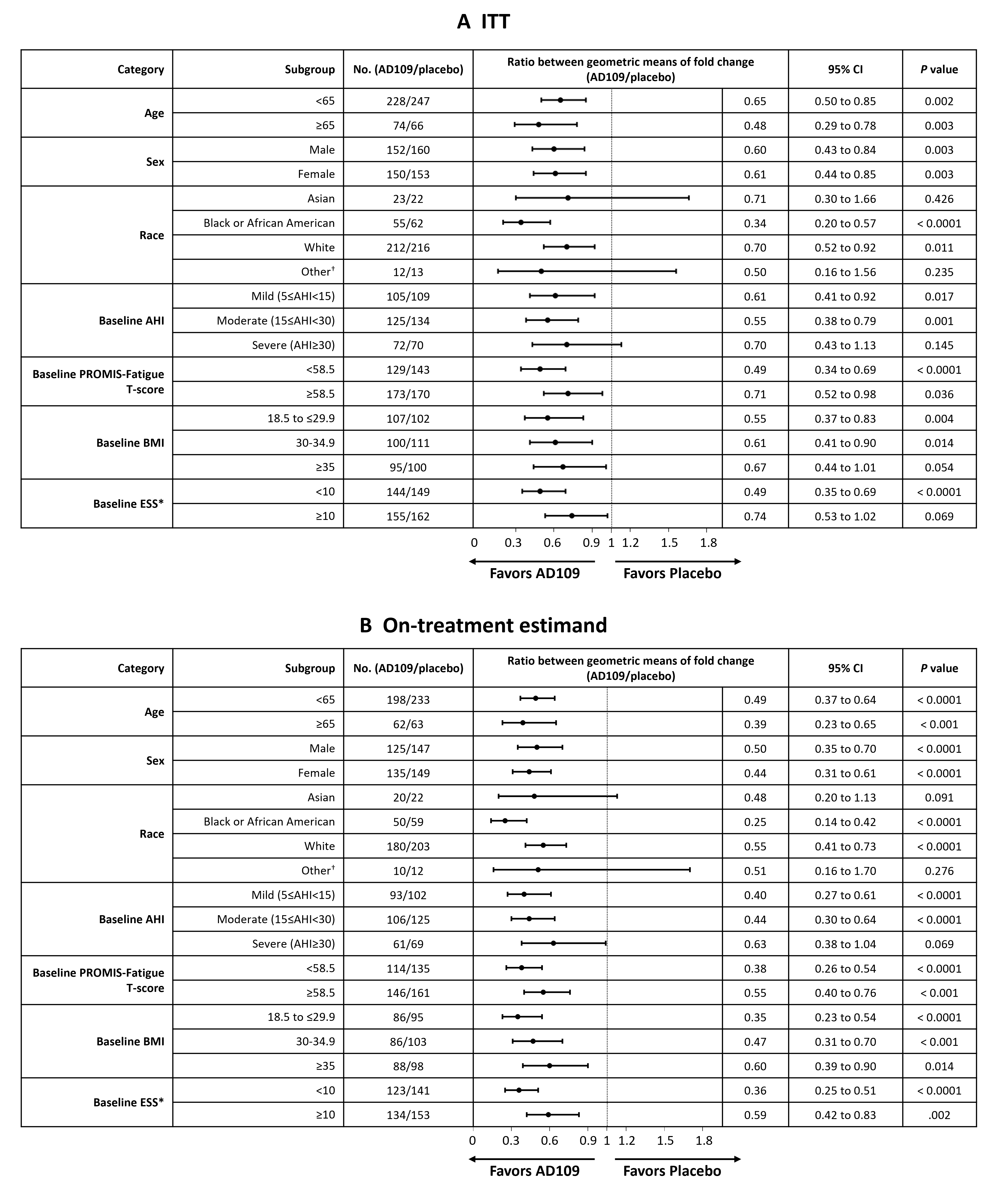

Supplement: aamag215_Supplementary_Data [file aamag215_supplementary_data.zip › Figure E5.png]

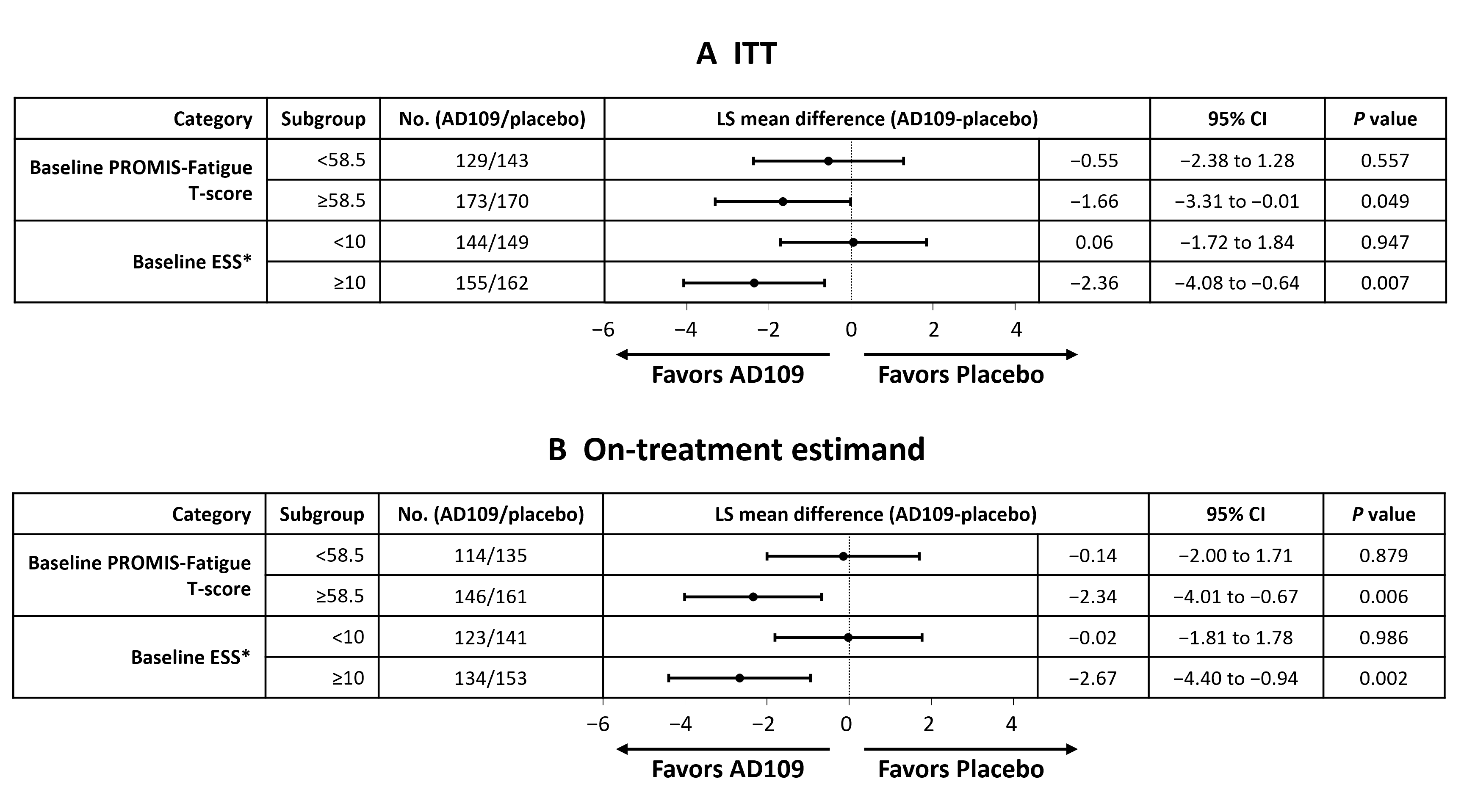

Supplement: aamag215_Supplementary_Data [file aamag215_supplementary_data.zip › Figure E6.png]

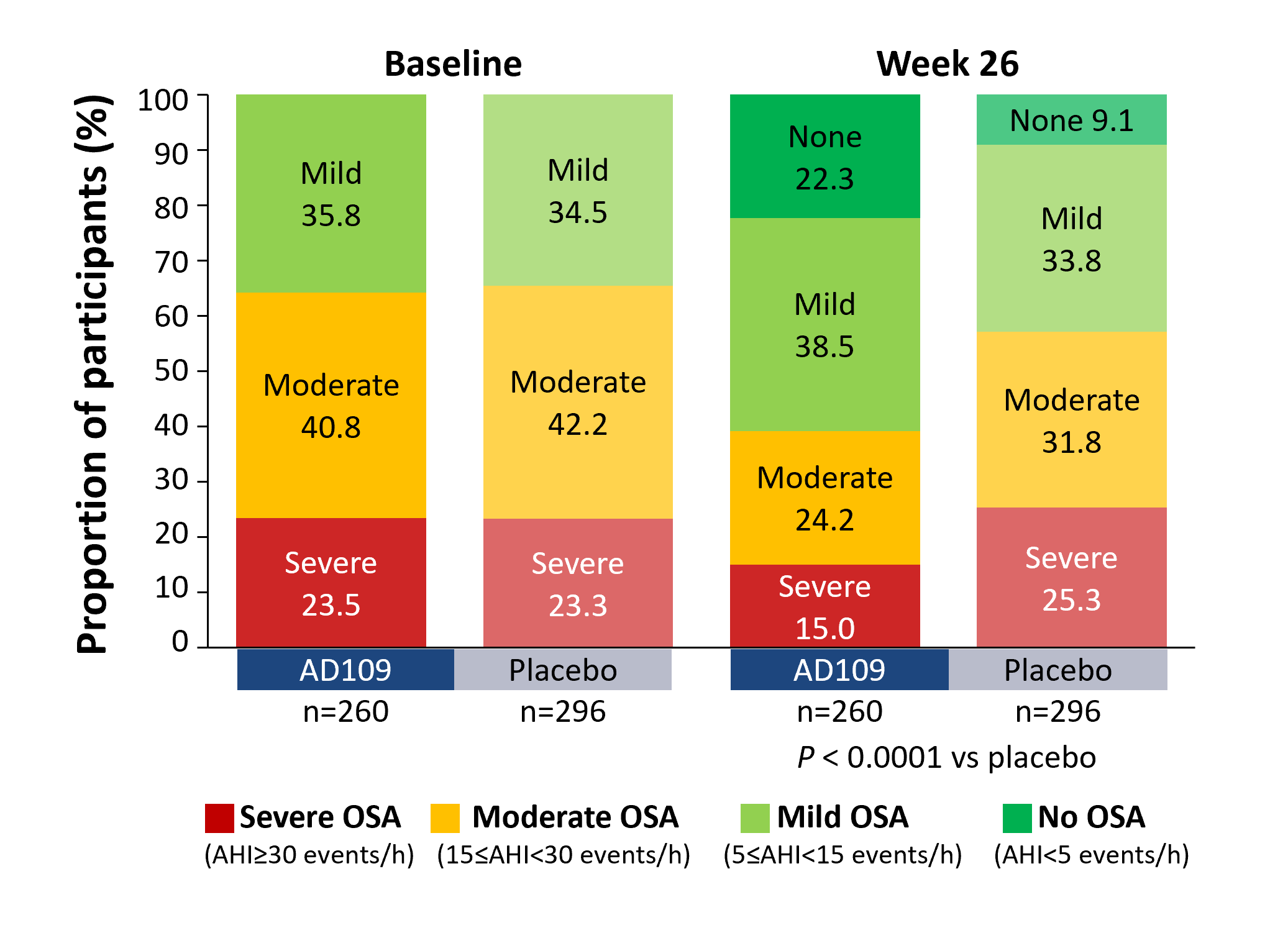

Supplement: aamag215_Supplementary_Data [file aamag215_supplementary_data.zip › Figure E7.png]

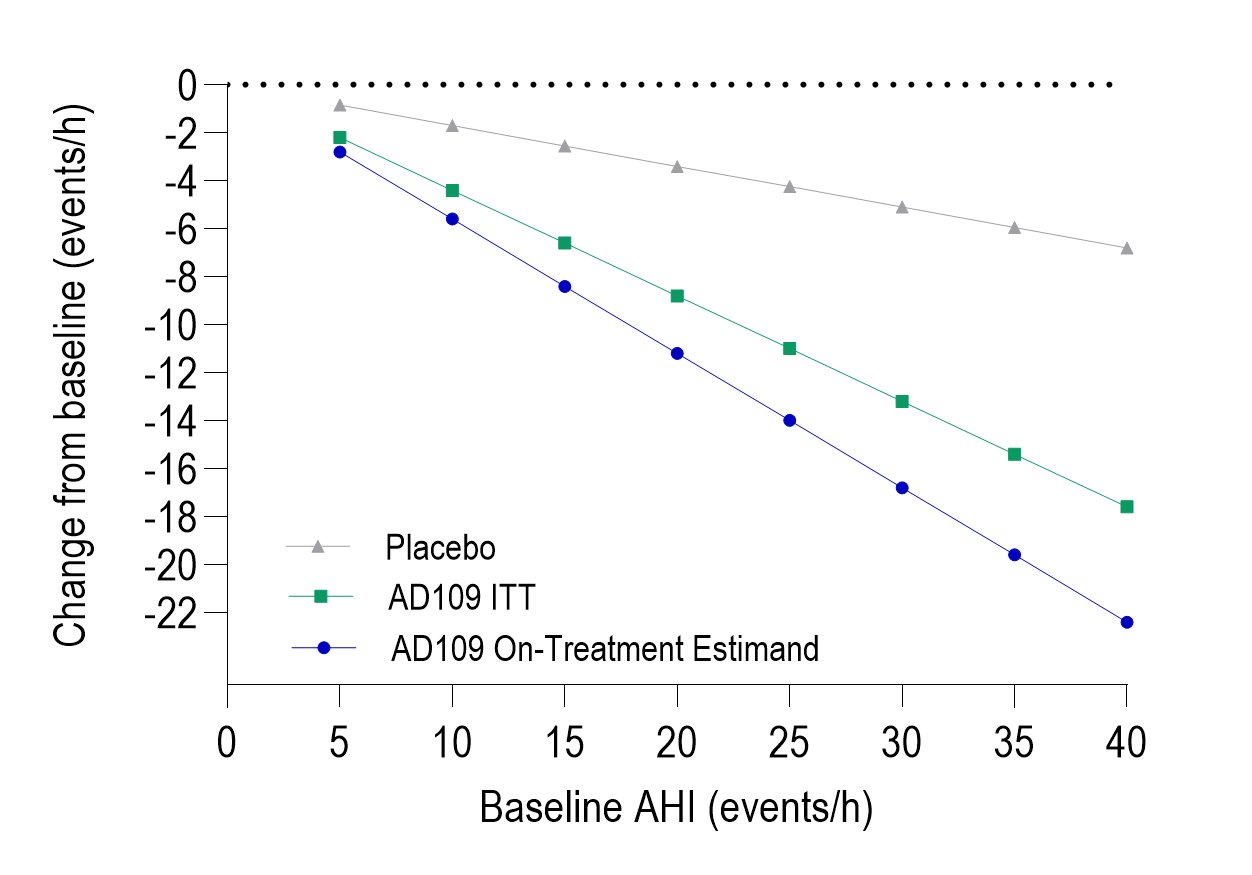

Supplement: aamag215_Supplementary_Data [file aamag215_supplementary_data.zip › Figure E8.png]

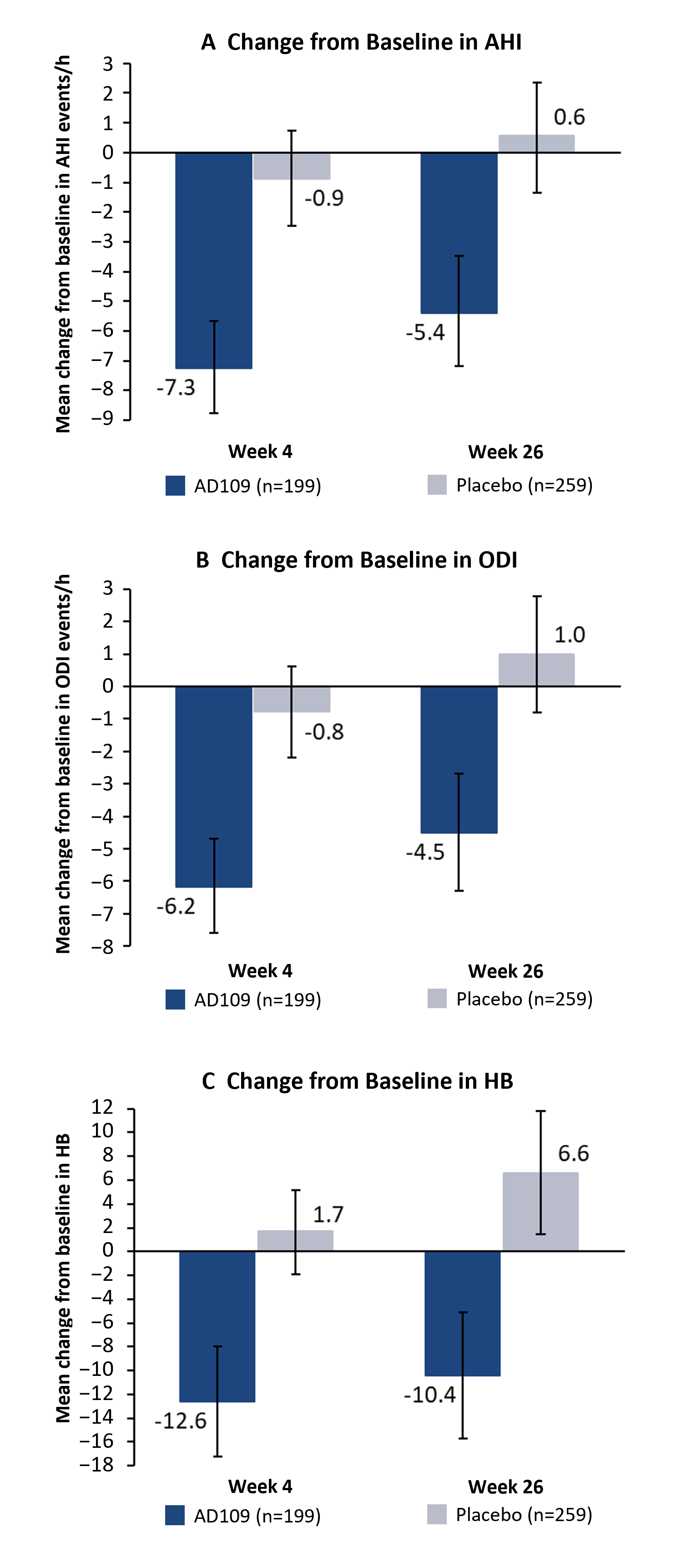

Supplement: aamag215_Supplementary_Data [file aamag215_supplementary_data.zip › Figure E9.png]

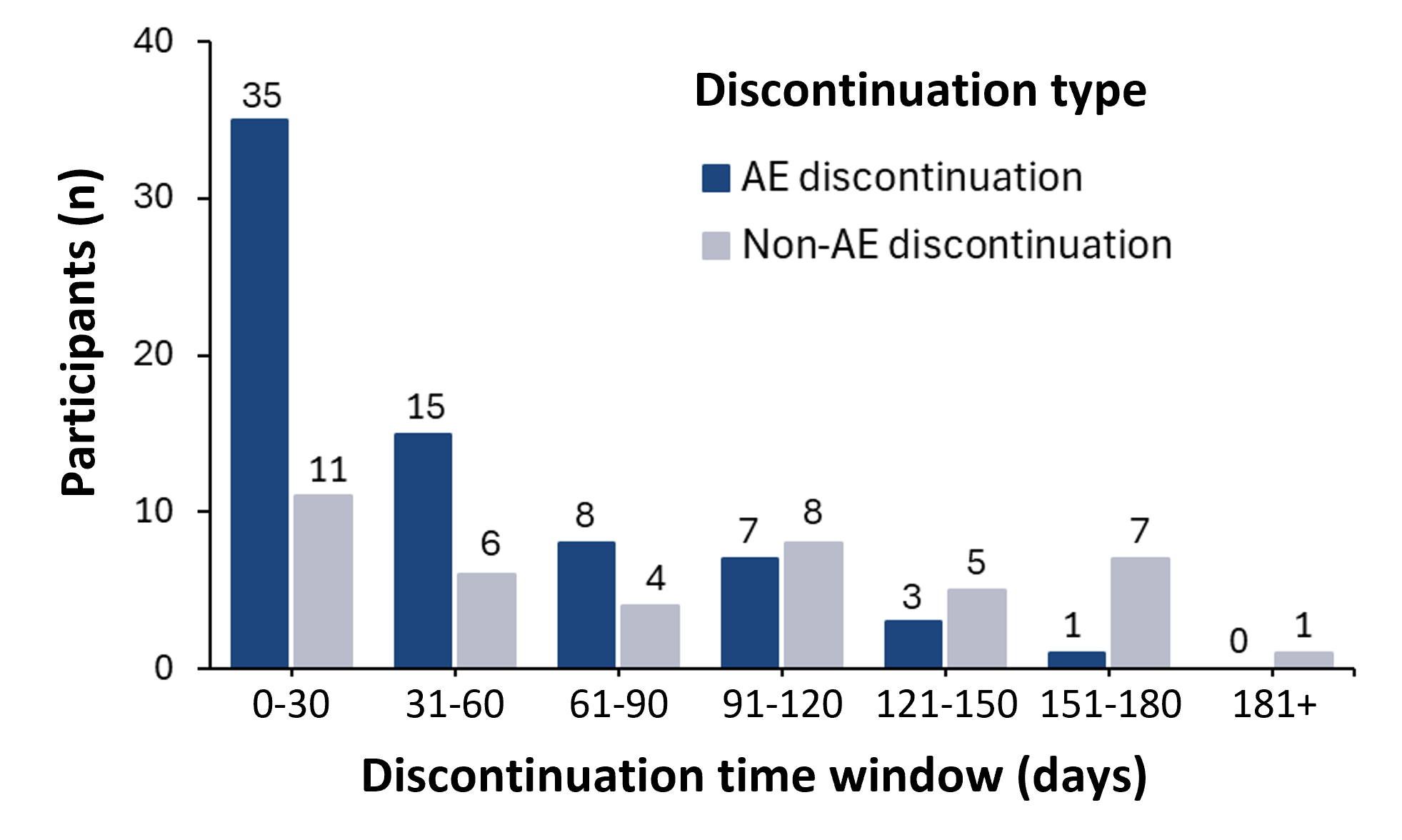

Supplement: aamag215_Supplementary_Data [file aamag215_supplementary_data.zip › Figure E10.png]
